# Ru(II)-based complexes containing 2-thiouracil derivatives suppress liver cancer stem cells by targeting NF-κB and Akt/mTOR signaling

**DOI:** 10.1038/s41420-024-02036-w

**Published:** 2024-06-03

**Authors:** Larissa M. Bomfim, Sara P. Neves, Amanda M. R. M. Coelho, Mateus L. Nogueira, Rosane B. Dias, Ludmila de F. Valverde, Clarissa A. G. Rocha, Milena B. P. Soares, Alzir A. Batista, Rodrigo S. Correa, Daniel P. Bezerra

**Affiliations:** 1 Gonçalo Moniz Institute, Oswaldo Cruz Foundation (IGM-FIOCRUZ/BA), Salvador, Bahia 40296-710 Brazil; 2 Department of Propedeutics, School of Dentistry of the Federal University of Bahia, Salvador, Bahia 40110-909 Brazil; 3 Department of Biological Sciences, State University of Feira de Santana, Feira de Santana, Bahia 44036-900 Brazil; 4 Department of Dentistry, Federal University of Sergipe, Lagarto, Sergipe 49400-000 Brazil; 5 Department of Pathology, School of Medicine of the Federal University of Bahia, Salvador, Bahia 40110-909 Brazil; 6 Center for Biotechnology and Cell Therapy, D’Or Institute for Research and Education (IDOR), Salvador, Bahia 41253-190 Brazil; 7 SENAI Institute of Innovation (ISI) in Health Advanced Systems, University Center SENAI/CIMATEC, Salvador, Bahia 41650-010 Brazil; 8 Department of Chemistry, Federal University of São Carlos, São Paulo, São Carlos 13561-901 Brazil; 9 Department of Chemistry, Federal University of Ouro Preto, Ouro Preto, Minas Gerais 35400-000 Brazil

**Keywords:** Cancer stem cells, Pharmacology

## Abstract

Cancer stem cells (CSCs) are defined as a rare population of cancer cells related to tumor initiation and maintenance. These cells are primarily responsible for tumor growth, invasion, metastasis, recurrence, and resistance to chemotherapy. In this paper, we demonstrated the ability of Ru(II)-based complexes containing 2-thiouracil derivatives with the chemical formulas *trans-*[Ru(2TU)(PPh_3_)_2_(bipy)]PF_6_ (**1**) and *trans-*[Ru(6m2TU)(PPh_3_)_2_(bipy)]PF_6_ (**2**) (where 2TU = 2-thiouracil and 6m2TU = 6-methyl-2-thiouracil) to suppress liver CSCs by targeting NF-κB and Akt/mTOR signaling. Complexes **1** and **2** displayed potent cytotoxic effects on cancer cell lines and suppressed liver CSCs from HepG2 cells. Increased phosphatidylserine exposure, loss of mitochondrial transmembrane potential, increased PARP (Asp214) cleavage, DNA fragmentation, chromatin condensation and cytoplasmic shrinkage were detected in HepG2 cells treated with these complexes. Mechanistically, complexes **1** and **2** target NF-κB and Akt/mTOR signaling in HepG2 cells. Cell motility inhibition was also detected in HepG2 cells treated with these complexes. Complexes **1** and **2** also inhibited tumor progression in mice with HepG2 cell xenografts and exhibited tolerable systemic toxicity. Taken together, these results indicate that these complexes are new anti-HCC drug candidates that can suppress liver CSCs.

## Introduction

Liver cancer is one of the leading causes of cancer mortality worldwide. In 2020, 905,700 people were diagnosed with liver cancer, and 830,200 people died worldwide. By 2040, the incidence of new cases and fatalities from liver cancer might increase by >55% [1, 2].

The most frequent type of primary liver cancer is hepatocellular carcinoma (HCC). For patients with advanced HCC who are not candidates for resection, ablation, or transplantation but who have retained liver function, systemic chemotherapy is the primary therapeutic option [3, 4]. Sorafenib, an oral multikinase inhibitor, was the first drug approved to treat advanced HCC. Although sorafenib extends patient survival by only a few months, it helps to stabilize the tumor [5]. Recently, the combination of atezolizumab and bevacizumab has increased the number of available HCC treatment options [6–8]. However, new drug options are still urgently needed.

Cancer stem cells (CSCs) are defined as a small and elusive subset of cancer cells that can initiate and sustain tumor growth [9–11]. These cells are primarily responsible for tumor growth, invasion, metastasis, recurrence, and resistance to chemotherapy. Extensive research has been performed on CSCs to determine surface markers and cellular signaling pathways governing the CSC phenotype as potential targets to eliminate CSCs [9–11].

**Fig. 1:**
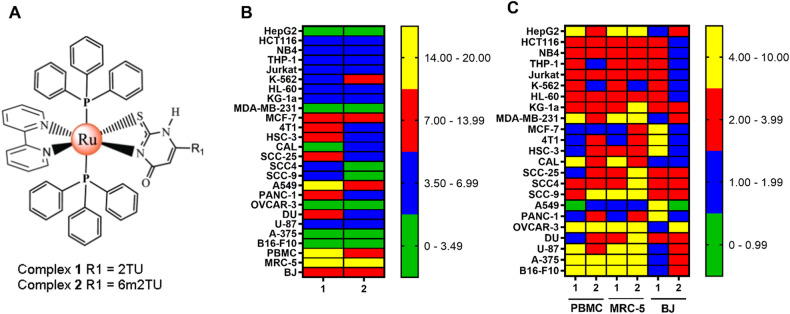
Ru(II) complexes containing 2-thiouracil derivatives exhibit potent and selective cytotoxic effects on cancer cells. **A** Chemical structures of complexes **1** and **2**. **B** Heatmap of the IC_50_ values in μM of cytotoxicity of complexes **1** and **2** against cancer and noncancerous cells obtained by the Alamar Blue assay after 72 h of incubation. **C** Heatmap of selectivity indices (SIs) obtained for complexes **1** and **2**. The data were calculated using the following formula: SI = IC_50_ [noncancerous cells]/IC_50_ [cancer cells].

Ruthenium complexes have been reported to be potential anti-HCC agents [12–14]. Recently, we synthesized two cytotoxic Ru(II)-based complexes containing 2-thiouracil derivatives with the chemical formulas *trans-*[Ru(2TU)(PPh_3_)_2_(bipy)]PF_6_ (**1**) and *trans-*[Ru(6m2TU)(PPh_3_)_2_(bipy)]PF_6_ (**2**), where 2TU = 2-thiouracil and 6m2TU = 6-methyl-2-thiouracil (Fig. 1A) [15]. In the present work, we demonstrated the ability of complexes **1** and **2** to effectively suppress liver CSCs through targeted inhibition of the NF-κB and Akt/mTOR signaling pathways.

## Results

### Ru(II) complexes containing 2-thiouracil derivatives display potent cytotoxicity in cancer cell lines

The cytotoxic effects of complexes **1** and **2** were evaluated on a panel of 23 cancer cell lines (HepG2, HCT116, NB4, THP-1, Jurkat, K-562, HL-60, KG-1a, MDA-MB-231, MCF-7, 4T1, HSC-3, CAL 27, SCC-25, SCC4, SCC-9, A549, PANC-1, OVCAR-3, DU 145, U-87 MG, A-375, and B16-F10) and three noncancerous cells (PBMC, MRC-5, and BJ) using the Alamar Blue assay after 72 h of treatment (Fig. 1B and Table S1). Both complexes displayed potent cytotoxicity against all cancer cell lines, with half-maximal inhibitory concentration (IC_50_) values ranging from 2.4 μM in OVCAR-3 ovarian cancer cells to 17.5 μM in A549 lung cancer cells for complex **1** and from 1.6 μM in OVCAR-3 ovarian cancer cells to 10.5 µM in A549 lung cancer cells for complex **2**. Doxorubicin was used as a positive control and showed cytotoxicity in all cell lines.

In noncancerous cells, complex **1** had an IC_50_ of 17.7 μM in MRC-5 pulmonary fibroblasts, 7.0 μM in BJ foreskin fibroblasts and 14.2 μM in PBMCs. In comparison, complex **2** presented IC_50_ values of 15.2 μM in MRC-5 pulmonary fibroblasts, 7.8 μM in BJ foreskin fibroblasts and 11.7 μM in PBMCs. The selectivity indices (SIs) were calculated by the following formula: SI = IC_50_ ([noncancerous cells]/IC_50_ [cancerous cells]). Figure 1C and Table S2 present the calculated SI. Curiously, both complexes showed an SI > 2 for many of the cancer cells investigated.

To study the anti-HCC potential of these complexes, the HCC cell line HepG2 was used in further experiments. Therefore, the viability of HepG2 cells treated with complex **1** at concentrations of 2, 4, and 8 µM and complex **2** at concentrations of 1.5, 3, and 6 µM was determined by trypan blue assay after 12, 24, 48, and 72 h of incubation. Both complexes reduced HepG2 cell viability in a concentration- and time-dependent manner (Fig. S1A–1D). After 72 h of incubation, complex **1** reduced cell viability by 46.5, 72.2, and 95.8%, while complex **2** inhibited cell viability by 39.4, 61.4, and 93.5%, respectively.

### Ru(II) complexes containing 2-thiouracil derivatives suppress liver CSCs from HCC HepG2 cells

**Fig. 2:**
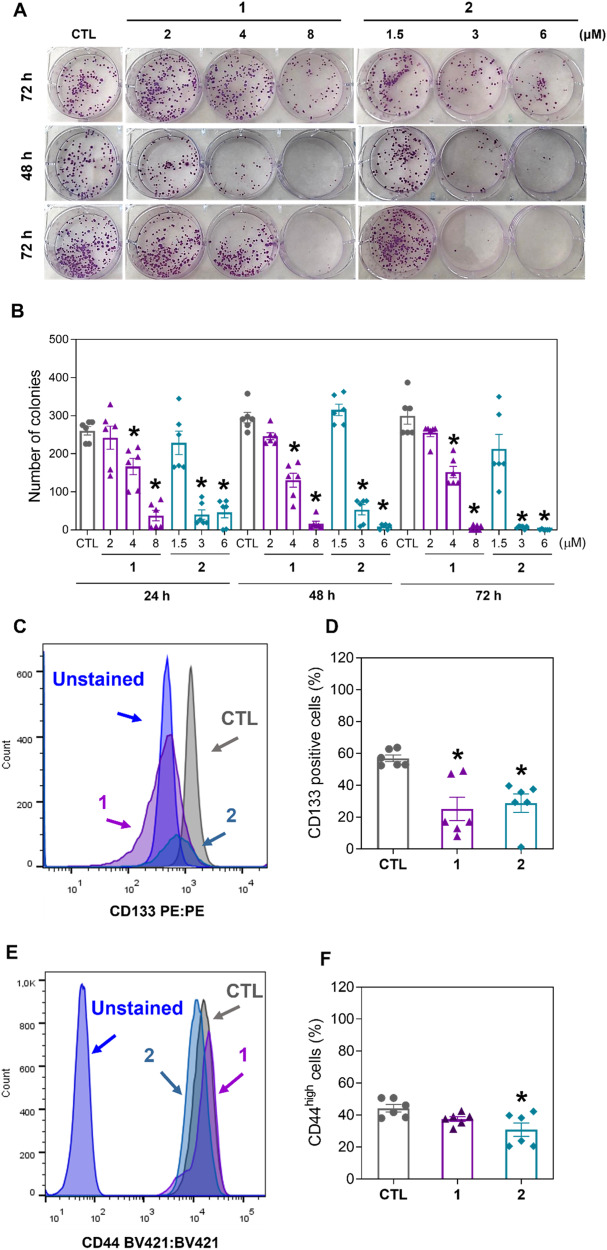
Ru(II) complexes containing 2-thiouracil derivatives suppress liver CSCs from HepG2 cells. **A** Representative images and (**B**) quantification of the number of colonies formed from HepG2 cells after treatment with complexes **1** and **2**. (**C** and **D**) Quantification of CD133 expression on HepG2 cells after 24 h of incubation with 8 μM complex **1** or 6 μM complex **2**, as determined by flow cytometric analysis. (**E** and **F**) Quantification of CD44^high^ in HepG2 cells after 24 h of incubation with 8 μM complex **1** or 6 μM complex **2**, as determined by flow cytometric analysis. The vehicle (0.2% DMSO) was used as a negative control (CTL). The data are expressed as the mean ± S.E.M. of three biological replicates carried out in duplicate. *P < 0.05 compared to CTL by one-way analysis of variance (ANOVA) followed by Dunnett’s multiple comparisons test.

To determine whether complexes **1** and **2** can act against liver CSCs, we first performed a long-term colony formation assay to determine whether these complexes affect the clonogenic ability of HCC HepG2 cells. Clonogenic assays are well-known methods for evaluating the stemness of CSCs since a single CSC can form clonogenic colonies [16, 17]. Interestingly, treatment with both complexes significantly decreased the clonogenic viability of HepG2 cells in a concentration- and time-dependent manner (Fig. 2A, B).

Next, we quantified the expression of two biomarkers of liver CSCs, CD133 [18] and CD44 [19], in HepG2 cells treated with complexes **1** and **2**. Likewise, both complexes reduced the percentage of HepG2 CD133-positive cells (Fig. 2C, D), while complex **2** reduced the percentage of HepG2 CD44^high^ cells (Fig. 2E, F).

In a new set of experiments, we measured the effects of complexes **1** and **2** on three-dimensional (3D) tumorspheres formed from HepG2 cells since multicellular 3D tumor spheroids are well-known cell culture systems that can enrich cells with CSC characteristics [20, 21]. Both complexes reduced HepG2 tumorsphere growth (Fig. S2 and S3) and caused cell death (Fig. S4), corroborating that these molecules may inhibit CSCs in HCC HepG2 cells.

### Ru(II) complexes containing 2-thiouracil derivatives cause apoptotic cell death in HCC HepG2 cells

A series of cellular and molecular analyses were performed to examine the mechanism of cell death in HepG2 cells treated with complexes **1** and **2**. HepG2 cells that were treated with complexes **1** and **2** for 24, 48, and 72 h showed cell morphology changes that were associated with apoptosis, including a reduction in cell volume, chromatin condensation, and fragmentation of the nuclei, as observed in May-Grunwald-Giemsa-stained cells (Fig. S5).

Light scattering characteristics measured by flow cytometry were used to analyze cellular parameters such as size and complexity/granularity in HepG2 cells treated with complexes **1** and **2** (Fig. S6A–S6F). Forward light scattering (FSC) was employed as a cell size metric in this experiment, while side scattering (SSC) was used to determine cell complexity/granularity. Treatment with these complexes caused cell shrinkage, as indicated by a decrease in the FSC, accompanied by an increase in the SSC, probably due to nuclear condensation. Both morphological changes are associated with cellular apoptosis, corroborating the findings observed in cells stained with May-Grunwald-Giemsa.

**Fig. 3:**
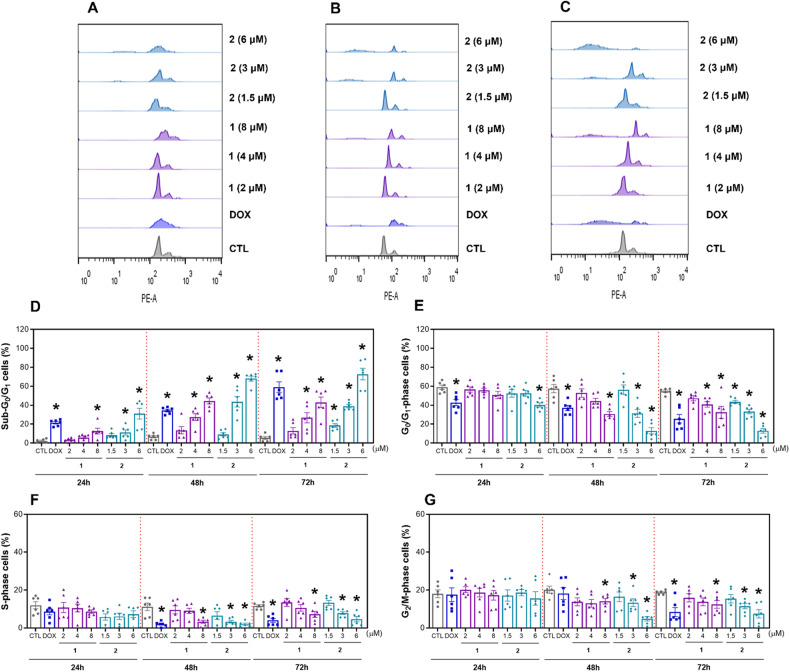
Effect of Ru(II) complexes containing 2-thiouracil derivatives on the DNA fragmentation and cell cycle distribution of HepG2 cells. Representative flow cytometric histograms of the cell cycle distribution of HepG2 cells after treatment with complexes **1** and **2** after 24 (**A**), 48 (**B**) and 72 (**C**) h of incubation. The percentages of cells in the sub-G_0_/G_1_ (**D**), G_0_/G_1_ (**E**), S (**F**) and G_2_/M (**G**) phases were quantified via flow cytometric analysis. The vehicle (0.2% DMSO) was used as a negative control (CTL), and doxorubicin (DOX, 1 μM) was used as a positive control. The data are expressed as the mean ± S.E.M. of three biological replicates carried out in duplicate. *P < 0.05 compared to CTL by one-way analysis of variance (ANOVA) followed by Dunnett’s multiple comparisons test.

Internucleosomal DNA fragmentation and cell cycle distribution were evaluated in HepG2 cells after 24, 48, and 72 h of incubation with complexes **1** and **2** via a DNA content-based flow cytometry assay (Fig. 3A–G). All DNA of subdiploid size (sub-G_0_/G_1_) was considered fragmented. Both complexes induced DNA fragmentation in a time- and concentration-dependent manner. After 72 h of incubation, complex **1**, at concentrations of 2, 4, and 8 µM, caused DNA fragmentation by 12.3, 26.7, and 43.1%, respectively, while complex **2**, at concentrations of 1.5, 3, and 6 µM, induced DNA fragmentation by 18.6, 39.1, and 72.9%, respectively (against the 5.6% detected in the control). The cell cycle phases G_0_/G_1_, S and G_2_/M decreased proportionally in HepG2 cells treated with complexes **1** and **2**. Doxorubicin, used as a positive control, also caused DNA fragmentation.

**Fig. 4:**
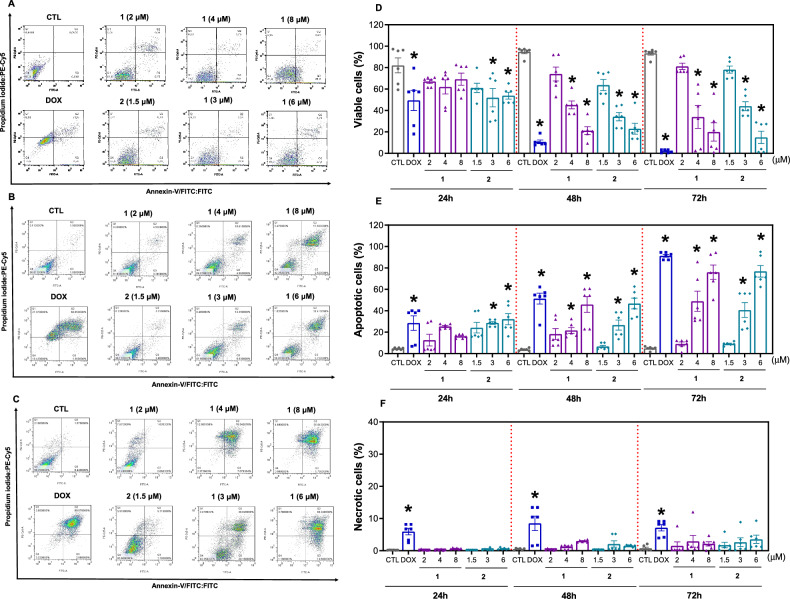
Ru(II) complexes containing 2-thiouracil derivatives cause apoptosis in HepG2 cells. Representative flow cytometric dot plots of HepG2 cells stained with annexin V-FITC/PI after treatment with complexes **1** and **2** after 24 (**A**), 48 (**B**) and 72 (**C**) h of incubation. The percentages of viable (annexin V-FITC^-^/PI^-^ cells) (**D**), apoptotic (early apoptotic [annexin V-FITC^+^/PI^-^ cells] plus late apoptotic [annexin V-FITC^+^/PI^+^ cells]) (**E**) and necrotic (annexin V-FITC^-^/PI^+^ cells) (**F**) cells were quantified. The vehicle (0.2% DMSO) was used as a negative control (CTL), and doxorubicin (DOX, 1 μM) was used as a positive control. The data are expressed as the mean ± S.E.M. of three biological replicates carried out in duplicate. *P < 0.05 compared to CTL by one-way analysis of variance (ANOVA) followed by Dunnett’s multiple comparisons test.

Annexin V-FITC/propidium iodide (PI) double staining was also applied to HepG2 cells treated with complexes **1** and **2** for 24, 48 and 72 h to quantify phosphatidylserine exposure and cell membrane integrity, which are markers of apoptosis and necrosis, respectively. Both complexes induced a significant increase in the percentage of apoptotic cells in a time- and concentration-dependent manner, and no significant increase in the percentage of necrotic cells was detected (Fig. 4A–F). After 72 h of incubation, complex **1**, at concentrations of 2, 4, and 8 µM, increased apoptosis by 9.0, 48.9, and 76.1%, respectively, while complex **2**, at concentrations of 1.5, 3, and 6 µM, increased apoptosis by 9.1, 40.7, and 76.9%, respectively (against 4.8% found in the control). Treatment with doxorubicin, which was used as a positive control, also led to apoptosis.

**Fig. 5:**
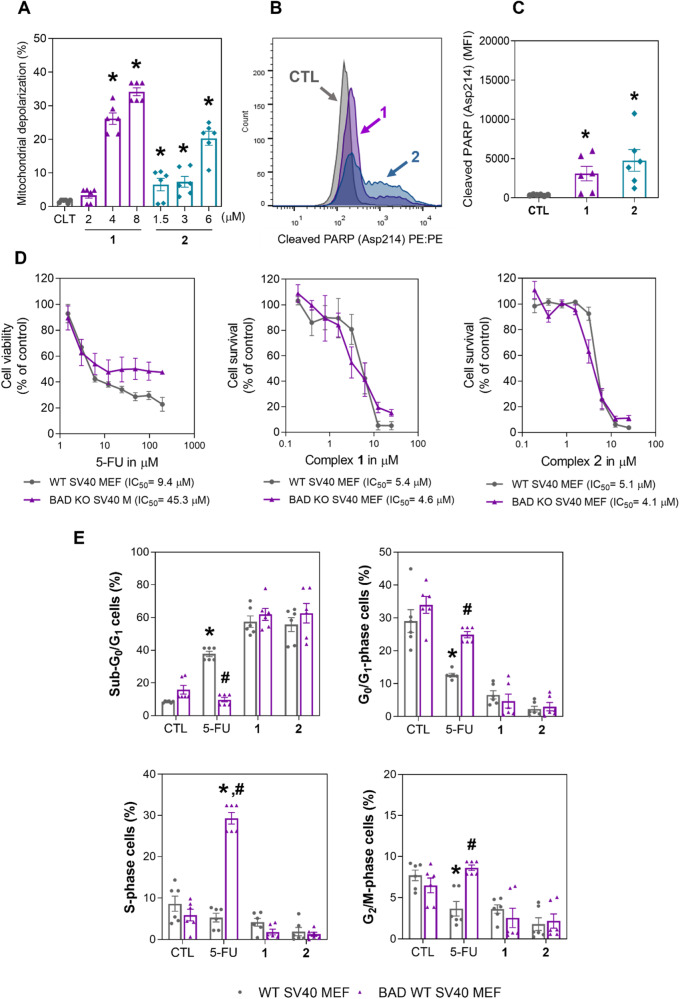
Ru(II) complexes containing 2-thiouracil derivatives induce cell death via mitochondrial dysfunction. **A** Quantification of mitochondrial membrane depolarization in HepG2 cells after 24 h of incubation with complex **1** or **2**, as determined by flow cytometry. **B** and **C** Quantification of PARP (Asp214) cleavage in HepG2 cells after 24 h of incubation with complex **1** (8 μM) or **2** (6 μM), as determined by flow cytometric analysis. MFI: Mean fluorescence intensity. **D** Survival curves of WT SV40 MEFs and BAD KO SV40 MEFs upon treatment with complexes **1** and **2** and 5-fluorouracil (5-FU, used as a positive control). The curves were obtained from at least three biological replicates carried out in duplicate using the Alamar Blue assay after 72 h of incubation. **E** DNA fragmentation (sub-G_0_/G_1_ cells) and cell cycle distribution (G_0_/G_1_, S and G_2_/M phases) of WT SV40 MEFs and BAD KO SV40 MEFs after 48 h of incubation with complexes **1** (10 μM) and **2** (10 μM) or 5-FU (40 μM). The vehicle (0.2% DMSO) was used as a negative control (CTL). The data are expressed as the mean ± S.E.M. of three biological replicates carried out in duplicate. *P < 0.05 compared to CTL by one-way analysis of variance (ANOVA) followed by Dunnett’s multiple comparisons test.

As mitochondrial dysfunction and PARP cleavage are well-known events in apoptotic cell death, mitochondrial transmembrane potential and PARP (Asp214) cleavage were also determined by flow cytometry. Significant mitochondrial depolarization (Fig. 5A) and increased levels of PARP (Asp214) cleavage (Fig. 5B, C) were found in HepG2 cells treated with complexes **1** and **2**, corroborating that these complexes can cause cell death via apoptosis. Moreover, the BAD KO SV40 MEF cell line, as well as its parental cell line, WT SV40 MEF, were used to assess the involvement of the proapoptotic protein BAD in the cell death caused by complexes **1** and **2** (Fig. 5D, E). On the other hand, these complexes cause cell death independent of the protein BAD.

### Ru(II) complexes containing 2-thiouracil derivatives target NF-κB and Akt/mTOR signaling in HCC HepG2 cells

**Fig. 6:**
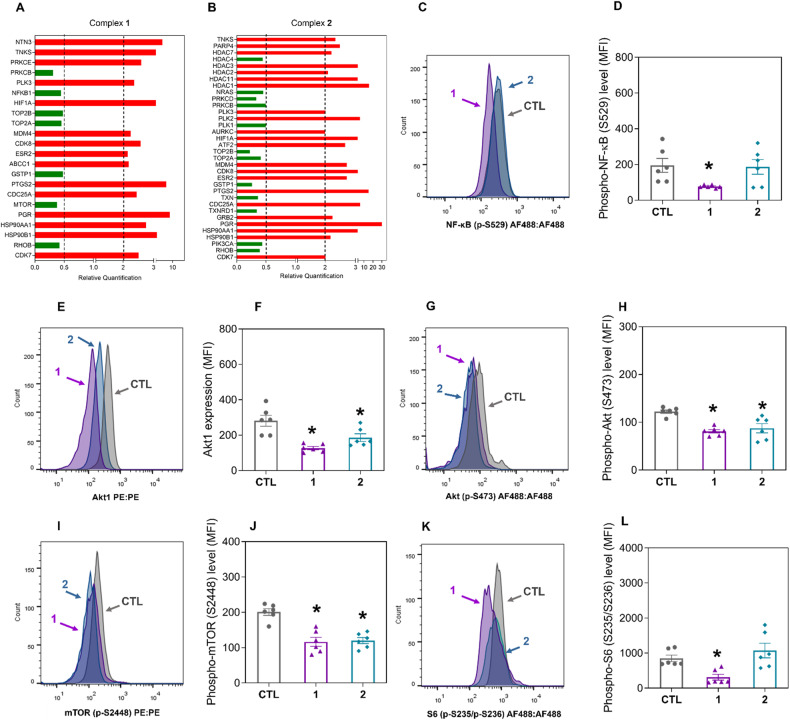
Ru(II) complexes containing 2-thiouracil derivatives affect NF-κB and Akt/mTOR signaling in HepG2 cells. **A**, **B** Genes up- and downregulated in HepG2 cells after 12 h of treatment with complexes **1** (8 μM) and **2** (6 μM). The vehicle (0.2% DMSO) was used as a negative control (CTL). The data are expressed as the relative quantification (RQ) compared to the CTL data. The genes were upregulated if RQ ≥ 2 (red bars) and downregulated if RQ ≤ 0.5 (green bars). Quantification of the levels of phospho-NF-κB p65 (S529) (**C**, **D**), Akt1 (**E**, **F**), phospho-Akt (S473) (**G**, **H**), phospho-mTOR (S2448) (**I**, **J**), and phospho-S6 (S235/S236) (**K**, **L**) in HepG2 cells after 24 h of incubation with complexes **1** (8 μM) and **2** (6 μM), as determined by flow cytometry. The vehicle (0.2% DMSO) was used as a negative control (CTL). The data are expressed as the mean ± S.E.M. of three biological replicates carried out in duplicate. *P < 0.05 compared to CTL by one-way analysis of variance (ANOVA) followed by Dunnett’s multiple comparisons test. MFI: Mean fluorescence intensity.

To investigate the molecular mechanism of action of complexes **1** and **2**, we analyzed the transcripts of 82 target genes using a qPCR array (Fig. 6A, B and Table S3). Among the altered gene transcripts, genes related to NF-κB (the *NFKB1* gene with RQ = 0.45 for complex **1**), PI3K/Akt/mTOR (the *PIK3CA* gene with RQ = 0.44 for complex **2**; the *MTOR* gene with RQ = 0.39 for complex **1**) and oxidative stress (the *GSTP1* gene with RQ = 0.49 for complex **1** and RQ = 0.27 for complex **2**; the *TXN* gene with RQ = 0.37 for complex **2**; and the *TXNRD1* gene with RQ = 0.35 for complex **2**) were downregulated in HepG2 cells treated with complexes **1** and **2**.

Next, the protein levels of several elements of the NF-κB and Akt/mTOR signaling pathways were quantified. The levels of phospho-NF-κB p65 (S529) (Fig. 6C, D), Akt1 (Fig. 6E, F), phospho-Akt (S473) (Fig. 6G, H), phospho-mTOR (S2448) (Fig. 6I, J), and phospho-S6 (S235/S236) (Fig. 6K, L) were reduced in complex **1**-treated HepG2 cells. In contrast, the levels of Akt1 (Fig. 6E, F), phospho-Akt (S473) (Fig. 6G, H), and phospho-mTOR (S2448) (Fig. 6I, J) were reduced after treatment with complex **2**, indicating that these complexes interfere with NF-κB and Akt/mTOR signaling. The levels of phospho-PI3K p85/p55 (T458/T199) (Fig. S7A and S7B), phospho-Akt (T308) (Fig. S7C and S7D), phospho-4EBP1 (T36/T45) (Fig. S7E and S7F), and phospho-elF4E (S209) (Fig. S7G and S7H) were not affected by treatment with these complexes.

As complexes **1** and **2** downregulate the level of phospho-mTOR (S2448), a negative regulator of autophagy [22], the effect of these complexes on the induction of autophagy was investigated. On the other hand, none of them caused autophagy, as assessed by quantification of p62/SQSTM1 expression levels in HepG2 cells treated with complexes **1** and **2** (Fig. S8A–S8C).

### Ru(II) complexes containing 2-thiouracil derivatives reduce the migration of HCC HepG2 cells

**Fig. 7:**
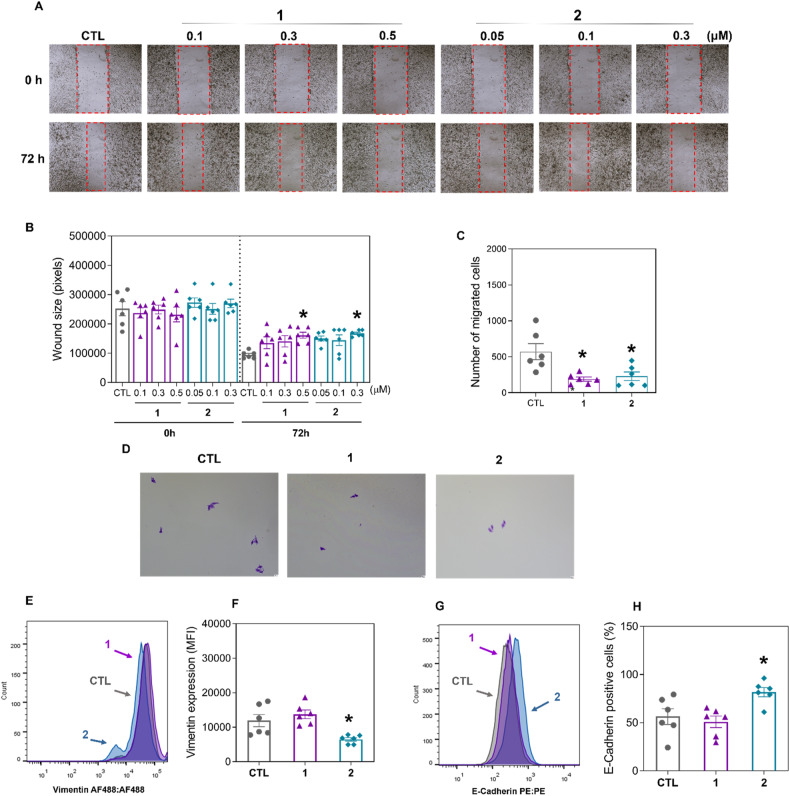
Ru(II) complexes containing 2-thiouracil derivatives interfere with the migration of HepG2 cells. **A** Representative images and (**B**) quantification of HepG2 cell migration in the wound healing assay after 72 h of incubation with complexes **1** and **2**. **C** Representative images and (**D**) quantification of HCT116 cell migration in the transwell migration assay after 24 h of incubation with complexes **1** (8 μM) and **2** (6 μM). Quantification of vimentin (**E**, **F**) and E-cadherin (**G**, **H**) expression in HepG2 cells after 24 h of incubation with complexes **1** (8 μM) and **2** (6 μM), as determined by flow cytometry. The vehicle (0.2% DMSO) was used as a negative control (CTL). The data are expressed as the mean ± S.E.M. of three biological replicates carried out in duplicate. *P < 0.05 compared to CTL by one-way analysis of variance (ANOVA) followed by Dunnett’s multiple comparisons test. MFI: Mean fluorescence intensity.

Since both complexes reduced the proportion of liver CSC markers in HepG2 cells and liver CSCs are directly associated with cell migration and invasion [23], we hypothesized that these complexes could reduce HepG2 cell motility. Initially, noncytotoxic concentrations of complexes **1** and **2** were selected (Fig. S9) and tested in the wound healing assay. Both complexes reduced HepG2 cell migration after 72 h of incubation at noncytotoxic concentrations (0.5 μM for complex **1** and 0.3 μM for complex **2**) (Fig. 7A, B). Similarly, both complexes, at the same concentrations, also reduced motility in a transwell cell migration assay (Fig. 7C, D) using HepG2 cells.

Next, the epithelial–mesenchymal transition (EMT) markers vimentin and E-cadherin were evaluated in HepG2 cells treated with complexes **1** and **2** after 24 h of incubation. Vimentin (Fig. 7E, F) was reduced, and E-cadherin (Fig. 7G, H) was increased by treatment with complex **2**, indicating that this molecule can modulate EMT.

### Ru(II) complexes containing 2-thiouracil derivatives inhibit tumor progression in mice with HCC HepG2 cell xenografts

**Fig. 8:**
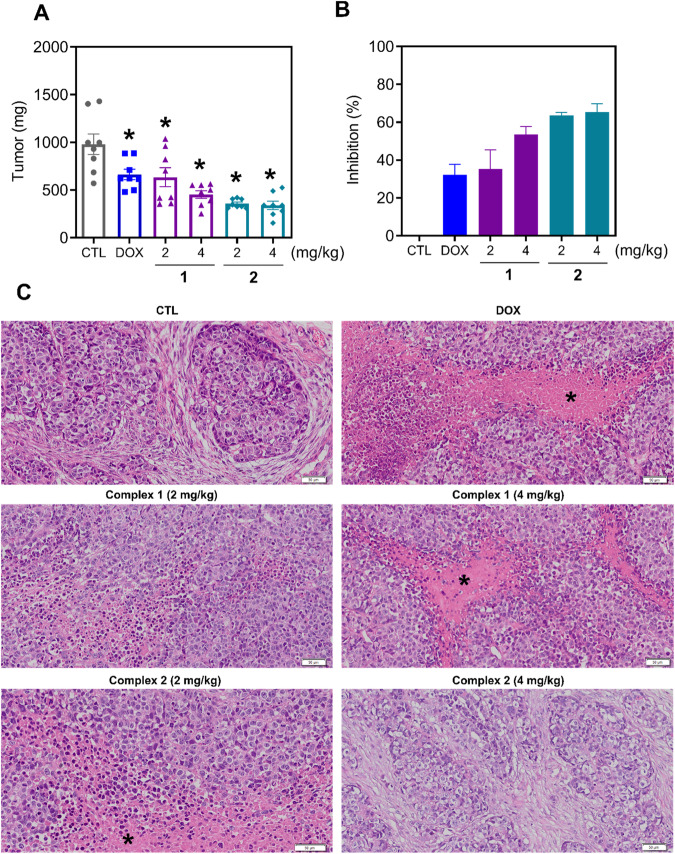
Ru(II) complexes containing 2-thiouracil derivatives inhibit tumor progression in mice with HepG2 cell xenografts. **A**, **B** In vivo antitumor activity of complexes **1** and **2** on C.B-17 SCID mice inoculated with HepG2 cells. The animals were treated with complexes **1** and **2** at doses of 2 or 4 mg/kg intraperitoneally once a day for 3 weeks. **C** Representative photomicrographs of HepG2 tumors from animals treated with complexes **1** and **2**. Histological sections were stained with hematoxylin-eosin and analyzed by light microscopy. The asterisks indicate areas of tissue necrosis. Scale bar = 50 μm. The vehicle (5% DMSO) was used as a negative control (CTL), and doxorubicin (DOX, 1 mg/kg) was used as a positive control. The data are expressed as the mean ± S.E.M. from 8 animals. *P < 0.05 compared to CTL by one-way analysis of variance (ANOVA) followed by Dunnett’s multiple comparisons test.

The in vivo antitumor activity of complexes **1** and **2** was investigated in C.B-17 SCID mice grafted with HepG2 cells. The animals were treated with 2 or 4 mg/kg of both complexes intraperitoneally once a day for 21 consecutive days. Both complexes inhibited the growth of HepG2 cells in mice (Fig. 8A, B). At the end of treatment, the mean tumor weight in the negative control group was 981 mg, while it was 665 mg in the doxorubicin-treated group. In complex **1**-treated animals, the mean tumor weights were 635 and 455 mg, corresponding to 35.3 and 53.6% tumor inhibition, respectively. In complex **2**-treated animals, the mean tumor weights were 358 and 340 mg, corresponding to 63.6 and 65.4% tumor inhibition, respectively. Doxorubicin reduced the tumor weight by 32.2%.

The tumors presented histological characteristics compatible with hepatocellular carcinoma, such as intense cellular and nuclear pleomorphism, hyperchromatism, atypical mitotic figures, and hepatocyte-like cells (Fig. 8C). The histological grading of the tumors varied from poorly to moderately differentiated in all the experimental groups. The tumor cells were organized in nodules or cords surrounded by a poorly vascularized collagen matrix. Areas of coagulative necrosis were frequent, especially in more central tumor regions. In addition, an infiltrate of inflammatory cells, predominantly mononuclear, was observed mainly adjacent to the necrotic areas. Areas of dystrophic calcification were observed in some of the tumors in the negative control, doxorubicin and complex **2** (4 mg/kg) groups. Furthermore, invasion fronts in the muscular tissue were observed in the control groups.

The toxicity parameters of animals treated with complexes **1** and **2** were also examined. No significant changes in body weight or organs (liver, kidney, lung, or heart) were detected in the animals treated with these complexes (*P* > 0.05) (Fig. S10A–S10F). Histopathological analysis of the kidneys (Fig. S11), livers (Fig. S12), and lungs (Fig. S13) of mice treated with complexes **1** and **2** revealed some alterations that were minor and/or reversible, indicating little damage to normal tissues. No significant changes were observed in the hearts of the animals treated with complexes **1** and **2** (data not shown).

## Discussion

Ru(II)-based organometallic complexes are an emerging class of potential antineoplastic drugs for the treatment of different types of cancer [24–29]. In this work, we report for the first time that two Ru(II) complexes, **1** and **2**, suppress liver CSCs by targeting NF-κB and Akt/mTOR signaling.

Previously, Elumalai et al. [30] reported a Ru(II) metallacycle that can eliminate glioma CSCs. The Ru(II) triazine complex has been reported to have the capacity to eradicate colorectal and breast CSCs [31]. Ru(II)-p-cymene complexes of mesalazine derivatives have been reported to suppress colorectal CSCs [32]. Likewise, a ruthenium complex with 5-fluorouracil and a ruthenium-xanthoxylin complex has been demonstrated to inhibit colorectal CSCs [33, 34], while a ruthenium complex with bipyridine and terpyridine ligands suppressed pancreatic CSCs [35]. Complexes **1** and **2** displayed potent cytotoxicity against different cancer cell histological types and had the ability to suppress liver CSCs in HepG2 cells. This is the first report on the ability of ruthenium complexes to reduce liver CSCs.

Molecular, biochemical and morphological correlates of apoptotic cell death, including increased phosphatidylserine exposure, loss of mitochondrial transmembrane potential, increased PARP (Asp214) cleavage, DNA fragmentation, chromatin condensation and cytoplasmic shrinkage, were detected in HepG2 cells treated with complexes **1** and **2**. Ru(II) polypyridyl complexes have been reported to induce apoptosis in HepG2 cells through the activation of caspase-3, the cleavage of PARP, the production of intracellular ROS and a reduction in the mitochondrial membrane potential [36]. Ru(II) complexed with xanthoxyline caused S-phase arrest and ERK1/2-mediated apoptosis in HepG2 cells via a p53-independent mechanism [12].

Mechanistically, complexes **1** and **2** target NF-κB and Akt/mTOR signaling in HepG2 cells. Interestingly, Noureldeen et al. [37] demonstrated that a Ru(III)-pyrimidine Schiff base interferes with Akt/mTOR and NF-κB signaling in HepG2 cells. Moreover, cyclometalated Ru(II) β-carboline complexes inhibited ERK and Akt signaling in HeLa cervical cancer cells [38]. Ru(II)-cyclopentadienyl-derived complexes have been reported to inhibit the MEK/ERK and PI3K/Akt signaling pathways in the colorectal cancer cell lines RKO and SW480 [39]. Similarly, a ruthenium complex with 5-fluorouracil inhibited Akt/mTOR signaling [33], and a ruthenium-xanthoxyline complex targeted the Hsp90 chaperone [34] in HCT116 colorectal cancer cells. Cyclometalated Ru(II)-isoquinoline complexes alter Akt/GSK-3β/Fyn signaling in cisplatin-resistant lung cancer A549/DDP cells [40].

Cell motility inhibition was also detected in HepG2 cells treated with complexes **1** and **2**. Previously, Ru(II) carbonyl complexes were reported to inhibit HepG2 cell migration and invasion in a concentration-dependent manner [41]. Polypyridyl Ru(II) complexes were also able to inhibit migration and invasion in melanoma A375 cells and breast cancer MDA-MB-231 cells [42].

Hu et al. [36] demonstrated that a Ru(II) polypyridyl complex inhibits HepG2 tumor development in mice. Similarly, Ru(II) complexes containing heterocyclic thioamidates have been reported to reduce the growth of HepG2 cells engrafted in C.B-17 SCID mice [13]. Herein, complexes **1** and **2** also inhibited tumor progression in mice with HCC HepG2 cell xenografts and tolerated systemic toxicity. These results indicate that complexes **1** and **2** are new anti-HCC drug candidates that can suppress liver CSCs.

## Materials and methods

### Synthesis of Ru(II) complexes

Ru(II) complexes **1** and **2** were synthesized using the complex precursor *cis, trans-*[RuCl_2_(PPh_3_)_2_(bipy)] and the ligands 2TU or 6m2TU, as previously described [15]. Both complexes were characterized by analytical techniques. Complexes **1** and **2** were dissolved in sterile dimethyl sulfoxide (DMSO, Synth, Diadema, SP, Brazil) in a 5 mg/mL stock solution (kept at −20 °C) and diluted with culture media to varying concentrations.

### Cell culture

In this study, a panel of 23 cancer cell lines, two noncancerous cell lines, one primary noncancerous cell, and one mutant cell line and its parental cell lines were used (as detailed in Table S4). The cells were cultured according to the manufacturer’s instructions for each cell line or the ATCC guidelines for animal cell culture [43]. Adherent cells were harvested using a 0.25% trypsin-EDTA solution (Sigma‒Aldrich Co.). All cell lines were cultured in flasks at 37 °C with 5% CO_2_ and subcultured every 3–4 days to maintain exponential growth. All cell lines were tested for mycoplasma using a mycoplasma staining kit (Sigma‒Aldrich).

### Cell viability assay

The Alamar Blue assay was used to measure cell viability, as previously described [44]. Adherent cells were seeded in 96-well plates at a density of 7 × 10^3^ cells per well, and suspended cells were seeded at 3 × 10^4^ cells per well. Drugs were added to each well after overnight incubation for adherent cells or immediately after seeding for suspension cells, and incubation continued for an additional 72 h. The positive control included doxorubicin (Laboratorio IMA S.A.I.C., Buenos Aires, Argentina). Four hours (for cell lines) or 24 h (for primary cultures) before the end of incubation, 20 μL of resazurin (30 μM) (Sigma‒Aldrich Co. St. Louis, MO, USA) was added to each well. The absorbance at 570 and 600 nm was measured using a SpectraMax 190 microplate reader (Molecular Devices, Sunnyvale, CA, USA).

### Trypan blue exclusion assay

The number of viable cells (unstained cells) and nonviable cells (trypan blue-stained cells) was counted by the trypan blue exclusion test. Briefly, 90 μL of cell suspension was selected, and 10 μL of trypan blue (0.4%) was added. A hemocytometer was used to fill the aliquot of the homogenized cell suspension, and the cells were counted under a light microscope.

### Colony-forming assay

A total of 500 cells were plated in 6-well plates with 4 mL of complete medium and treated with the drugs for 24, 48, or 72 h to determine clonogenic potential. The medium was subsequently replaced with fresh drug-free medium, and the plates were cultivated for 14 days. After that, the cells were fixed in methanol and stained with 0.5% crystal violet. Using an optical microscope (Nikon, TS100), the number of colonies with >50 cells was counted.

### HepG2 tumorspheres

HepG2 cells were seeded at a low cell density (1000 cells/well in 2 mL) in 24-well low-adhesion plates (Corning, USA) using serum-free DMEM-F12 supplemented with 20 ng/mL EGF (PeproTech, USA), 20 ng/mL bFGF (PeproTech, USA), and B27 supplement (Invitrogen, Carlsbad, CA, USA). After 5 days of incubation (to allow spheroid formation), the cells were treated with 20, 10, 5, 2.5 or 1.25 µM of each complex. The cells were photographed after 0, 24, and 48 h of treatment using an optical microscope (Leica, DMI8). In a new set of experiments, cells were treated with 10 µM complex **1** or 5 µM complex **2** for 48 h, stained with acridine orange (1 μg/mL) plus PI (1 μg/mL) and analyzed by a Leica TCS SP8 confocal microscope (Leica Microsystems, Wetzlar, HE, Germany).

### Flow cytometry assays

Protein expression was measured using primary antibodies conjugated to the specific fluorochromes listed in Table S5. To stain the cell surface proteins, the cells were washed with an incubation buffer (0.5% bovine serum albumin in PBS), and then antibodies were added and incubated for 1 h at room temperature. The cells were then washed with PBS, and the fluorescence signal was measured by flow cytometry. CD133 and CD44 were quantified in YO-PRO-1 (Sigma‒Aldrich Co.) negative cells.

To stain intracellular proteins, the cells were harvested and resuspended in 0.5-1 mL of 4% formaldehyde for 10 min at 37 °C. The tube was then placed on ice for 1 min, after which the cells were prechilled and permeabilized using a sequence of 100% (30 min) to 90% (30 min) methanol with gentle mixing. After washing with incubation buffer (0.5% bovine serum albumin in PBS), primary antibodies were added and incubated for 1 h at room temperature. Finally, the cells were washed with PBS, and flow cytometry was used to measure cell fluorescence.

Internucleosomal DNA fragmentation and cell cycle distribution were determined by a DNA content assay. The cells were stained with PI using a solution containing 0.1% Triton X-100, 2 µg/mL PI, 0.1% sodium citrate, and 100 μg/mL RNAse (all from Sigma‒Aldrich) and incubated in the dark for 15 min at room temperature [45].

For apoptosis detection, cell viability was quantified using Annexin V-FITC/PI (FITC Annexin V Apoptosis Detection Kit I, BD Biosciences, San Jose, CA, USA) according to the manufacturer’s instructions. The cells were also stained with rhodamine 123 (5 μg/mL, Sigma Aldrich Co.) for 15 min at 37 °C in the dark to assess the mitochondrial transmembrane potential [46]. After the cells were washed, they were incubated in saline for 30 min in the dark at 37 °C and analyzed by flow cytometry.

For all flow cytometry analyses, cellular fluorescence was measured using a BD LSRFortessa cytometer, BD FACSDiva software (BD Biosciences) and FlowJo software 10 (FlowJo LLC; Ashland, OR, USA). A minimum of 10,000 events were obtained per sample for intracellular staining, and a minimum of 30,000 events per sample were acquired for cell surface protein staining. Doublets were removed using FSC-A vs FCS-H and SCC-A vs SCC-H. Cellular debris was excluded from the analysis.

### May-Grunwald-Giemsa staining

Cells were grown on coverslips and stained with May-Grunwald-Giemsa. Subsequently, we employed light microscopy to examine and analyze morphological changes.

### qPCR array

Total RNA was extracted using the RNeasy Plus Mini Kit (Qiagen; Hilden, Germany) following the manufacturer’s instructions. A NanoDrop® 1000 spectrophotometer (Thermo Fisher Scientific, Waltham, Massachusetts, USA) was used to quantify and assess the purity of the RNA. Reverse transcription was performed using the Superscript VILOTM enzyme (Invitrogen Corporation; Waltham, MA, USA), and a TaqMan® array human cancer drug target 96-well plate, fast (ID RPRWENH, Applied BiosystemsTM, Foster City, CA, USA) was used for the gene expression study by qPCR (ABI ViiA7, Applied Biosystems instrument). The cycle conditions were 2 min at 50 °C, 10 min at 95 °C, and 40 cycles of 15 s at 95 °C and 1 min at 60 °C. All the experiments were conducted under DNase/RNase-free conditions. The 2^-ΔΔCT^ method [47] was used to calculate the relative quantification (RQ) of mRNA expression using Gene Expression SuiteTM Software (Applied BiosystemsTM). Cells treated with the negative control (0.2% DMSO) were used as a calibrator, and the RQs of the reference genes *GAPDH*, *UBC* and *RPLP0* were used to normalize the responses.

Genes were considered upregulated if the RQ was ≥2, indicating that gene expression in drug-treated cells was at least twofold greater than that in negative control-treated cells. Similarly, genes were considered downregulated when RQ ≤ 0.5, meaning that gene expression in drug-treated cells was at least half of that in negative control-treated cells.

### Immunofluorescence staining

The cells were grown on coverslips in 24-well plates and exposed to the drugs for 24 h. The cells were then washed twice with saline solution, permeabilized with 0.5% Triton X-100, treated with RNase (10 μg/mL), and incubated overnight with a primary fluorochrome-conjugated antibody (for antibody details, see Table S5). The next day, the cells were washed with saline solution and mounted using Fluoromount-G with DAPI (Invitrogen, Thermo Fisher Scientific). The cells were examined using a Leica TCS SP8 confocal microscope (Leica Microsystems, Wetzlar, HE, Germany).

### Wound healing assay

Wound healing assays were performed as previously described [48]. The cells were grown to 80–90% confluency in 12-well plates, and a wound was formed by dragging a plastic pipette tip over the cell surface. The remaining cells were washed three times with saline solution to eliminate cell debris before being cultured in serum-free media and treated with drugs. After 0 and 72 h of incubation, the migrating cells in front of the wound were photographed using an optical microscope (Nikon, TS 100). The wound area was calculated using ImageJ software from the National Institutes of Health (NIH, USA).

### Transwell migration assay

Transwell plates were used for the cell migration assay, as previously described [49]. The cells were initially incubated in serum-free media for 24 h. We used uncoated cell culture inserts in 6-well plates (8 m pore size; Corning, USA). The upper chamber received 1.5 mL of serum-free medium, whereas the bottom chamber received 2 mL of medium containing 20% FBS. Cotton swabs were used to remove cells that remained in the top chamber after 24 h. The cells on the bottom surface of the membrane were fixed in 4% paraformaldehyde and stained with 0.5% crystal violet. The cells were imaged and counted using an optical microscope (Leica, DMI8).

### HCC tumor growth xenograft model

A total of 48 C.B-17 SCID mice (male and female, 20–25 g) were obtained from the FIOCRUZ-BA animal facility (Salvador, Bahia, Brazil) in accordance with experimental methods authorized by the local Animal Ethics Committee (#22/2021). All mice were fed a standard pellet diet (food and water available ad libitum) and housed in an artificially lit room (12 h dark/light cycle).

HepG2 cells (10^7^ cells/500 μL) were implanted subcutaneously into the left front armpit of mice as previously described [12, 13]. The mice were administered intraperitoneally (200 μL/animal) once a day for 3 weeks. The animals were randomly divided into six groups: group 1 received vehicle (5% DMSO solution) (*n* = 8); group 2 received 1 mg/kg doxorubicin (*n* = 8); group 3 received complex **1** at a dose of 2 mg/kg (*n* = 8); group 4 received complex **1** at a dose of 4 mg/kg (*n* = 8); group 5 received complex **2** at a dose of 2 mg/kg (*n* = 8); and group 6 received complex **2** at a dose of 4 mg/kg (*n* = 8). The animals were euthanized with an anesthetic overdose (thiopental, 100 mg/kg) 1 day after treatment, and the tumors were removed, weighed, and processed for histological examination.

All animals were weighed at the start and end of the experiment to assess toxicological aspects. The animals were continuously examined for anomalies during the trial. The livers, kidneys, lungs, and hearts were removed and weighed before being preserved in 4% formaldehyde, dehydrated in a graded alcohol series, cleaned in xylene, and embedded in paraffin wax. The tissue was sliced into 5 μm thick slices, stained with hematoxylin and/or periodic acid-Schiff (liver and kidney), and histologically evaluated via optical microscopy.

### Statistical analysis

The results are expressed as the mean ± S.E.M. or as IC_50_ values with a 95% confidence interval of at least three independent experiments (biological replicates) carried out in duplicate (technical replicates). For statistical analyses, one-way analysis of variance (ANOVA) followed by Dunnett’s multiple comparisons test (*P* < 0.05) was applied using GraphPad Prism (Intuitive Software for Science; San Diego, CA, USA).

### Supplementary information

SUPPLEMENTAL MATERIAL

## Data Availability

Data will be made available from the corresponding author upon reasonable request.
