# Supplementary material for: Ru(II)-based complexes containing 2-thiouracil derivatives suppress liver cancer stem cells by targeting NF-κB and Akt/mTOR signaling
Source: Cell Death Discov. 2024 Jun 3;10:270. doi: 10.1038/s41420-024-02036-w (PMC11148080; doi:10.1038/s41420-024-02036-w)
Supplement: Supplementary file 1 — SUPPLEMENTAL MATERIAL [file 41420_2024_2036_MOESM1_ESM.pdf]

Supplementary Material

Ru(II)-based complexes containing 2-thiouracil derivatives suppress liver cancer stem cells by targeting NF-κB and Akt/mTOR signaling

Larissa M. Bomfim[1], Sara P. Neves[1], Amanda M. R. M. Coelho[1], Mateus L. Nogueira[1], Rosane B. Dias[1,2,3], Ludmila de F. Valverde[1,4], Clarissa A. G. Rocha[1,2,5,6], Milena B. P. Soares[1,7], Alzir A. Batista[8], Rodrigo S. Correa[9], Daniel P. Bezerra[1,*]

[1]Gonçalo Moniz Institute, Oswaldo Cruz Foundation (IGM-FIOCRUZ/BA), Salvador, Bahia, 40296-710, Brazil.

[2]Department of Propedeutics, School of Dentistry of the Federal University of Bahia, Salvador, Bahia, 40110-909, Brazil.

[3]Department of Biological Sciences, State University of Feira de Santana, Feira de Santana, Bahia, 44036-900, Brazil.

[4]Department of Dentistry, Federal University of Sergipe, Lagarto, Sergipe, 49400-000, Brazil.

[5]Department of Pathology, School of Medicine of the Federal University of Bahia, Salvador, 40110-909, Bahia, Brazil.

[6]Center for Biotechnology and Cell Therapy, D'Or Institute for Research and Education (IDOR), Salvador, 41253-190, Bahia, Brazil.

[7]SENAI Institute of Innovation (ISI) in Health Advanced Systems, University Center SENAI/CIMATEC, Salvador, Bahia, 41650-010, Brazil.

[8]Department of Chemistry, Federal University of São Carlos, São Carlos, São Paulo, 13561-901, Brazil.

[9]Department of Chemistry, Federal University of Ouro Preto, Ouro Preto, Minas Gerais, 35400-000, Brazil.

***Corresponding author:** D. P. Bezerra, E-mail: daniel.bezerra@fiocruz.br

Tel/Fax + 55 71 3176 2272.

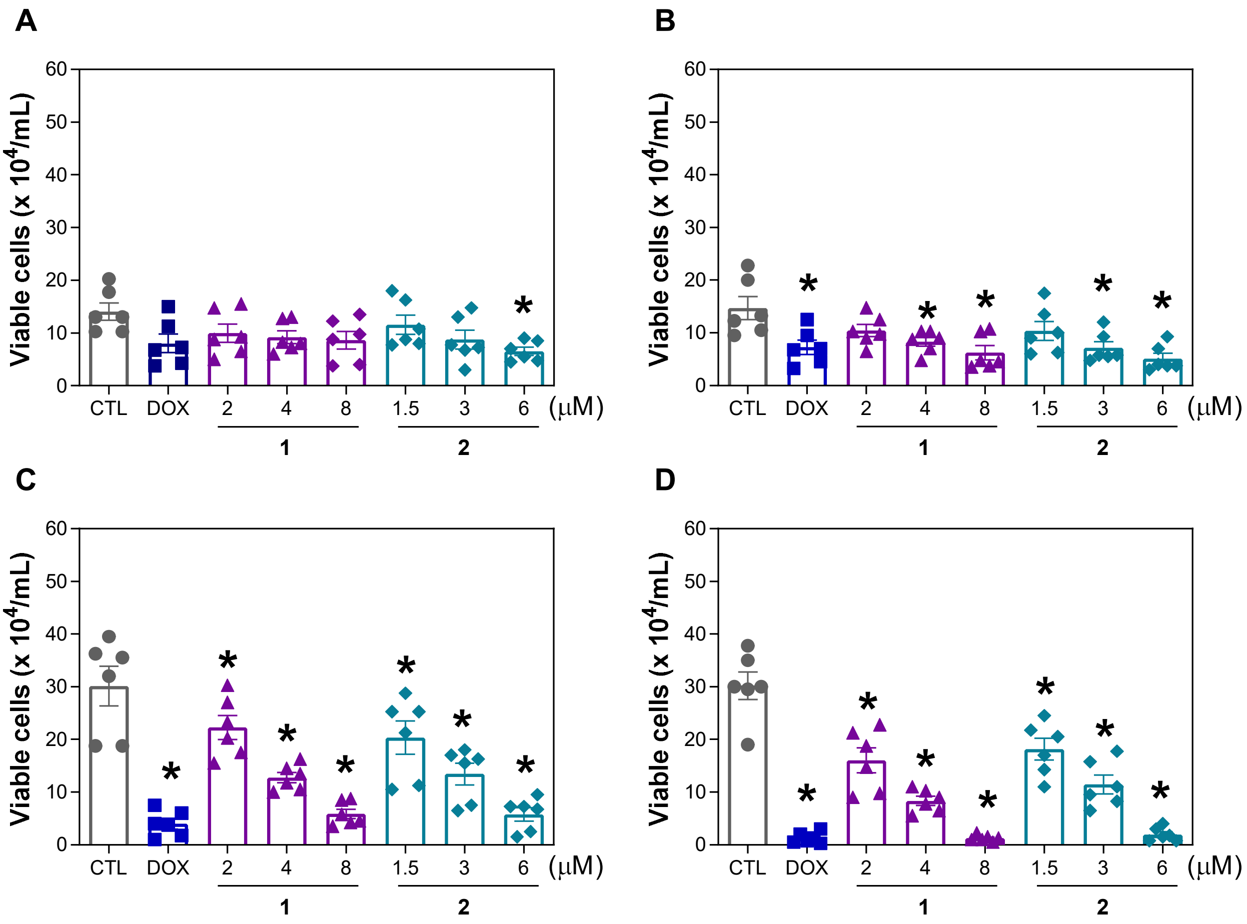

Figure S1. Quantification of viable HepG2 cells after 12 (**A**), 24 (**B**), 48 (**C**) and 72 (**D**) h of incubation with complexes **1** and **2**, as determined by the trypan blue method. The vehicle (0.2% DMSO) was used as a negative control (CTL), and doxorubicin (DOX, 1 μM) was used as a positive control. The data are expressed as the mean ± S.E.M. of three biological replicates carried out in duplicate. * $P < 0.05$ compared to CTL by one-way analysis of variance (ANOVA) followed by Dunnett's multiple comparisons test.

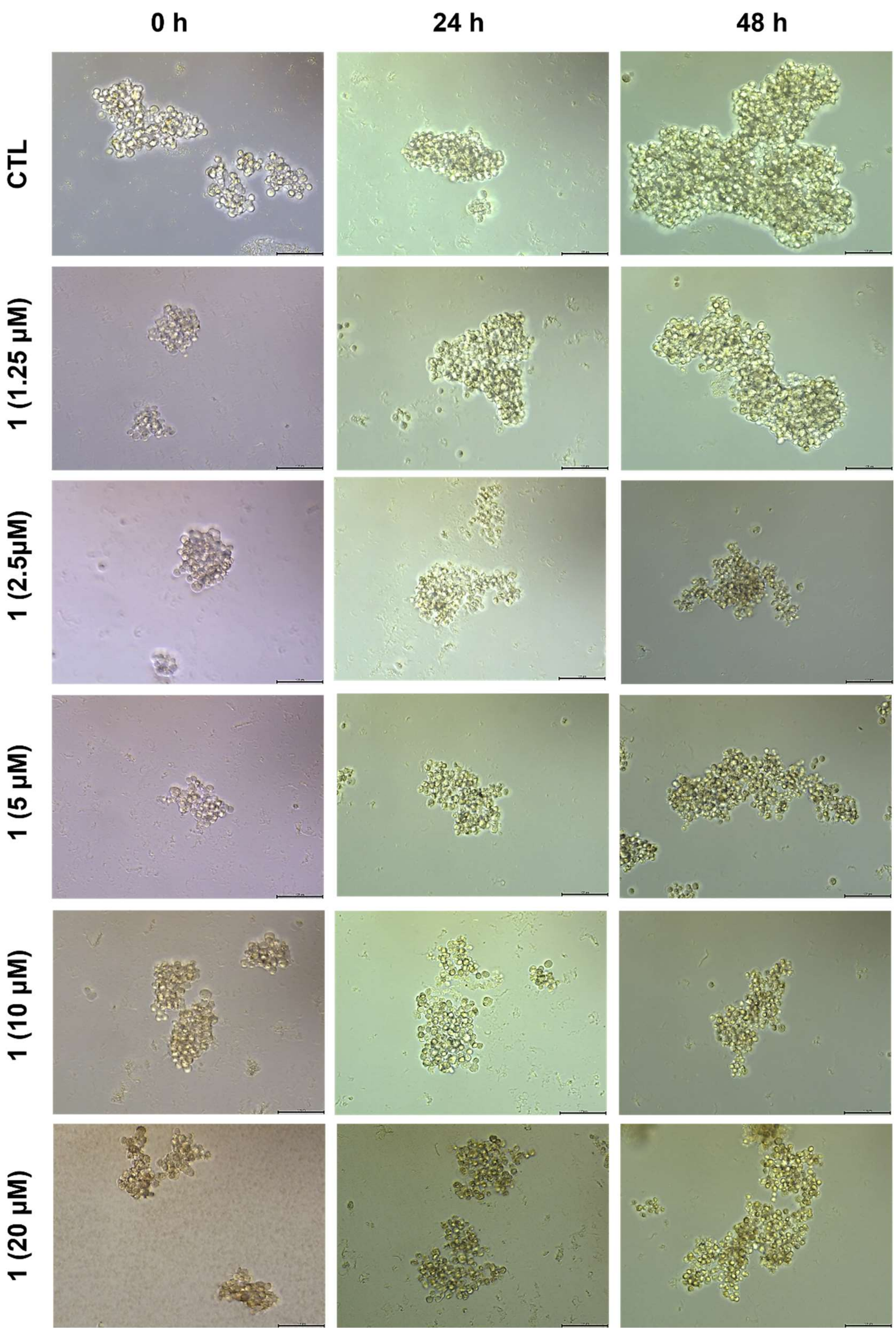

Figure S2. Representative light microscopy images of HepG2 tumorspheres treated with complex **1**. The vehicle (0.2% DMSO) was used as a negative control (CTL). Scale bar = 100 μm.

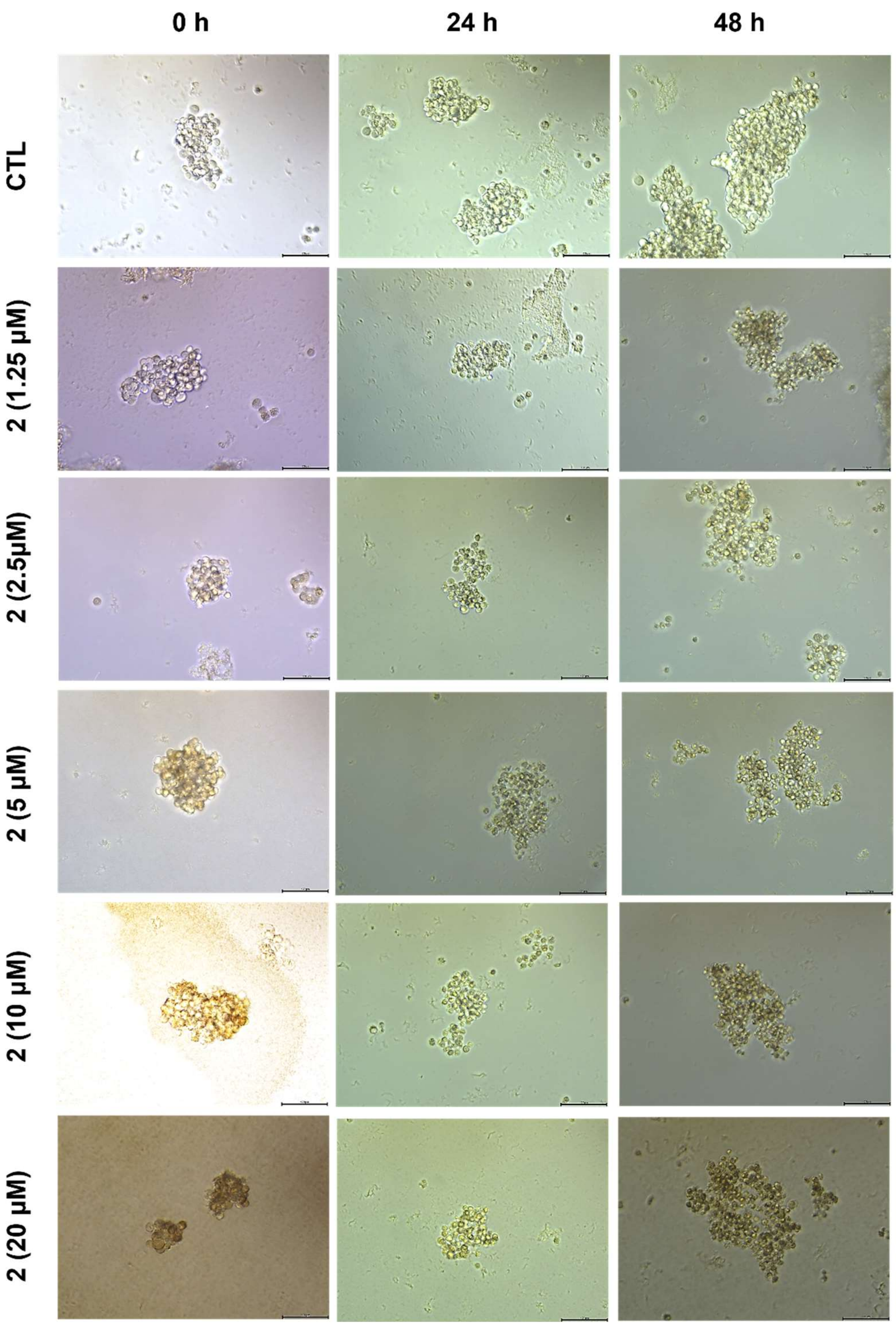

Figure S3. Representative light microscopy images of HepG2 tumorspheres treated with complex **2**. The vehicle (0.2% DMSO) was used as a negative control (CTL). Scale bar = 100 µm.

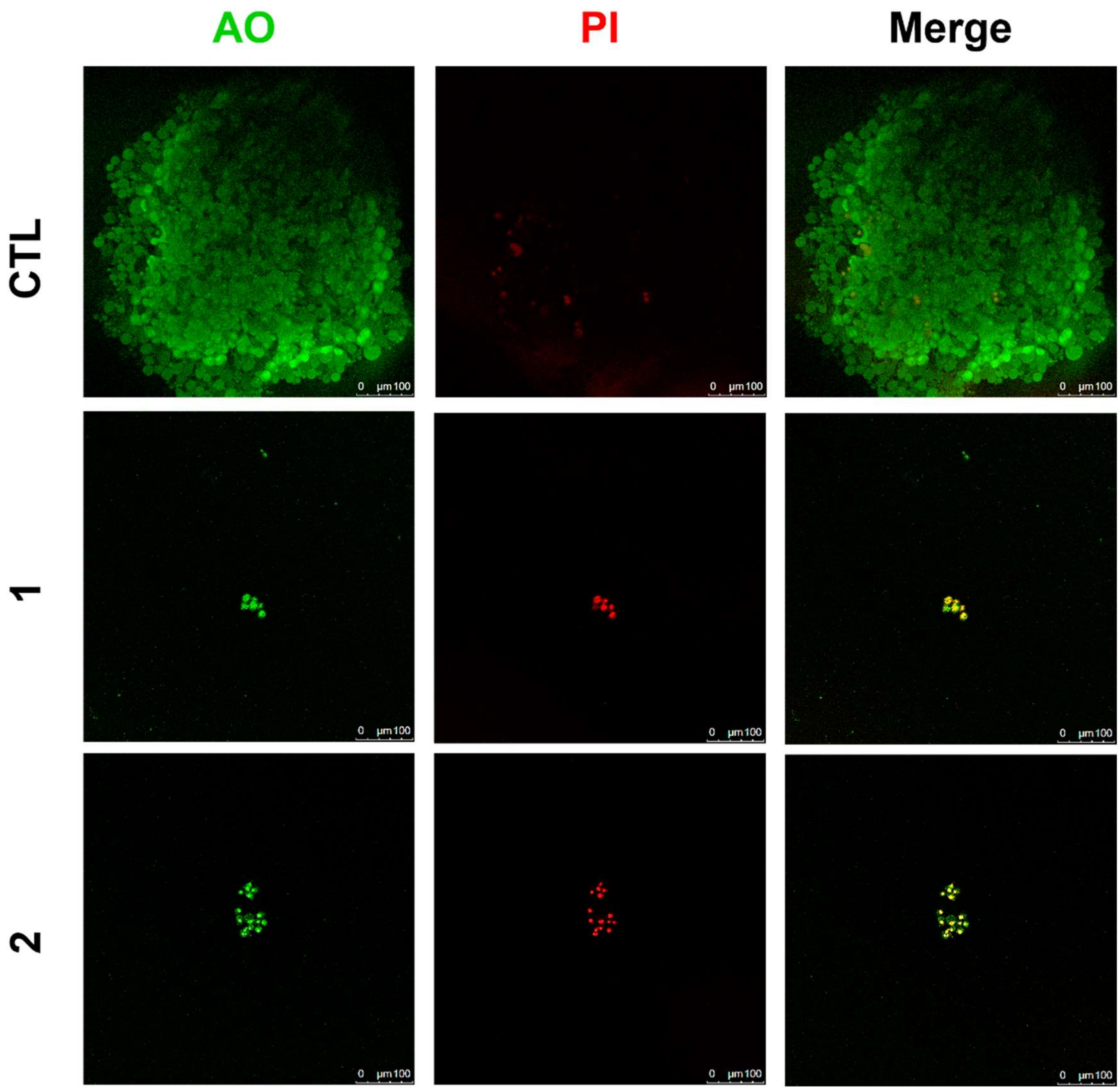

Figure S4. Representative confocal images of HepG2 tumorspheres treated with complexes **1** (10 μM) and **2** (5 μM) for 48 h of incubation. The cells were stained with acridine orange (AO, green cells) and propidium iodide (PI, red cells that represent dead cells). The vehicle (0.2% DMSO) was used as a negative control (CTL). Scale bar = 100 μm.

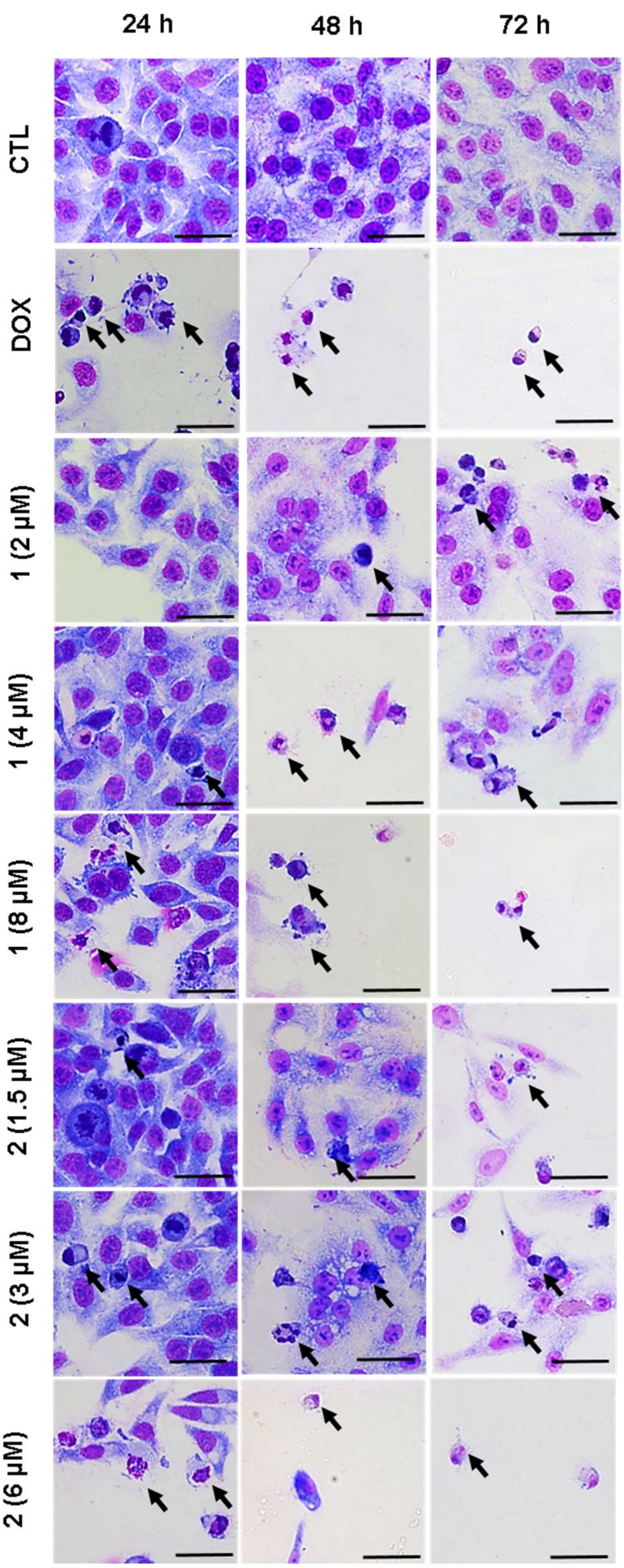

Figure S5. Effect of complexes **1** and **2** on the morphology of HepG2 cells, as assessed by May-Grunwald-Giemsa staining and examined by light microscopy (scale bar = 10 μm), after 24, 48 and 72 h of incubation. Vehicle (0.2% DMSO) was used as a negative control (CTL), and doxorubicin (DOX, 1 μM) was used as a positive control. Arrows indicate cells with chromatin condensation, fragmented DNA and/or cell shrinkage.

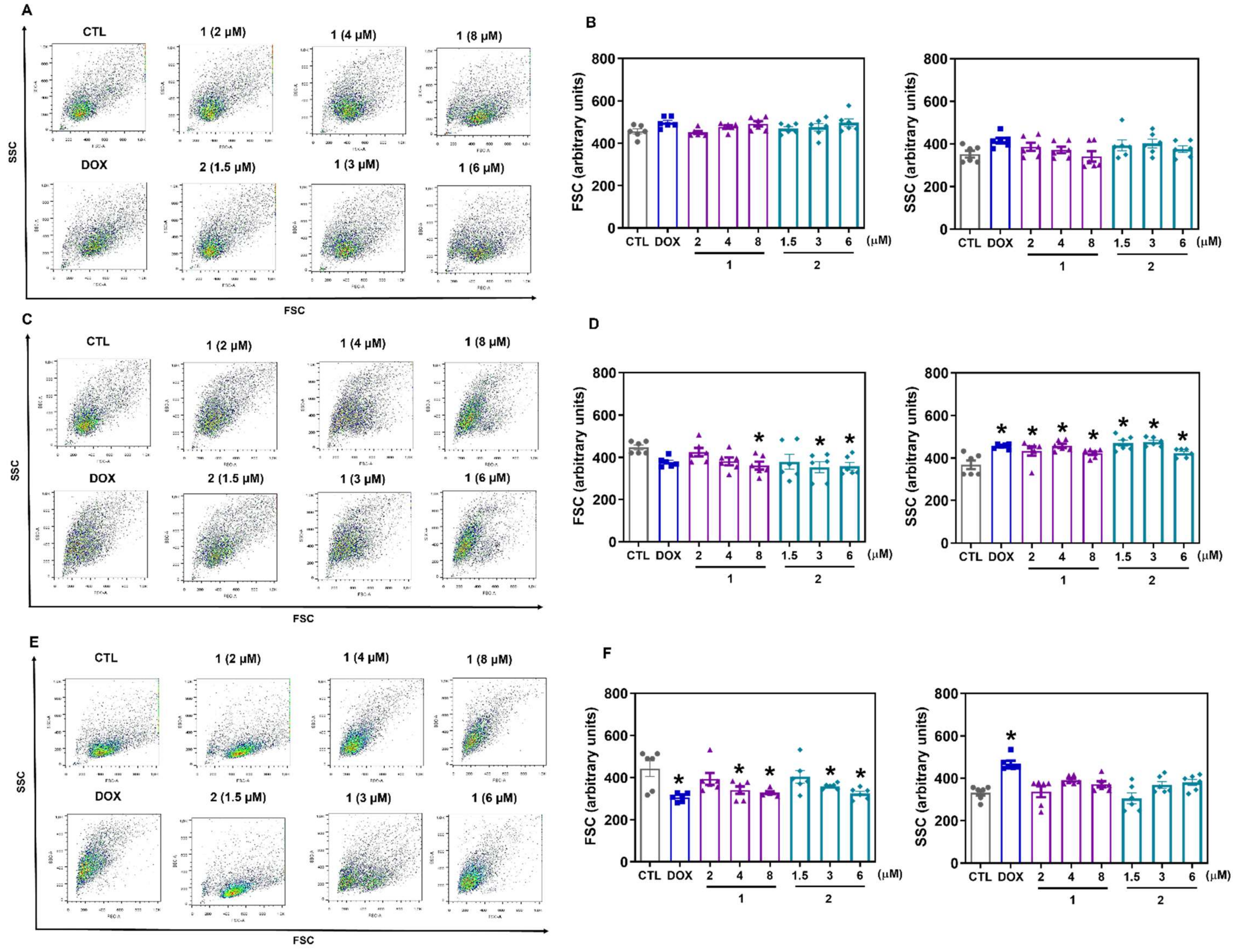

Figure S6. Effect of complexes **1** and **2** on the morphology of HepG2 cells, as assessed by the light-scattering features (forward scatter – FSC and side scatter – SSC) using flow cytometry after 24 (**A** and **B**), 48 (**C** and **D**) and 72 (**E** and **F**) h of incubation. The vehicle (0.2% DMSO) was used as a negative control (CTL), and doxorubicin (DOX, 1 μM) was used as a positive control. The data are expressed as the mean ± S.E.M. of three biological replicates carried out in duplicate. * $P < 0.05$ compared to CTL by one-way ANOVA followed by Dunnett's multiple comparisons test.

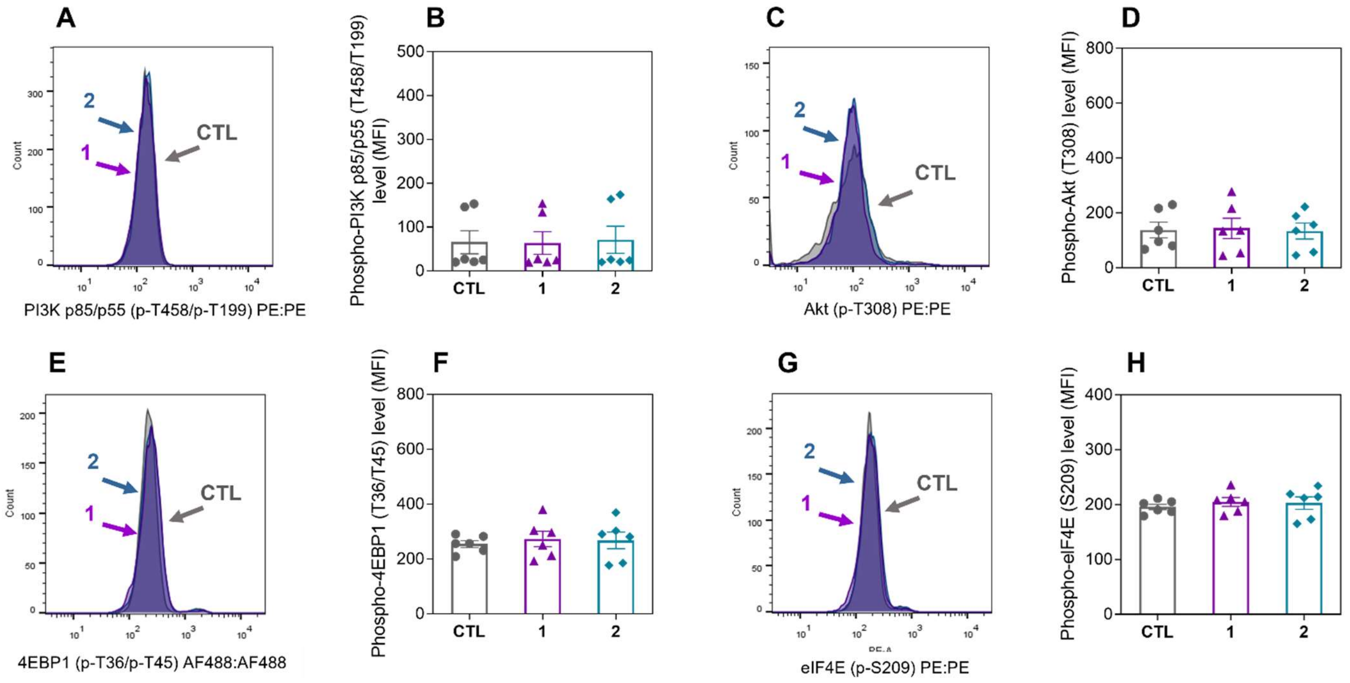

Figure S7. Quantification of the levels of phospho-PI3K p85/p55 (T458/T199) (**A** and **B**), phospho-Akt (T308) (**C** and **D**), phospho-4EBP1 (T36/T45) (**E** and **F**) and phospho-eIF4E (S209) (**G** and **H**) in HepG2 cells after 24 h of incubation with 8 μM complex **1** or 6 μM complex **2**, as determined by flow cytometric analysis. The vehicle (0.2% DMSO) was used as a negative control (CTL). The data are expressed as the mean ± S.E.M. of three biological replicates carried out in duplicate. MFI: Mean fluorescence intensity.

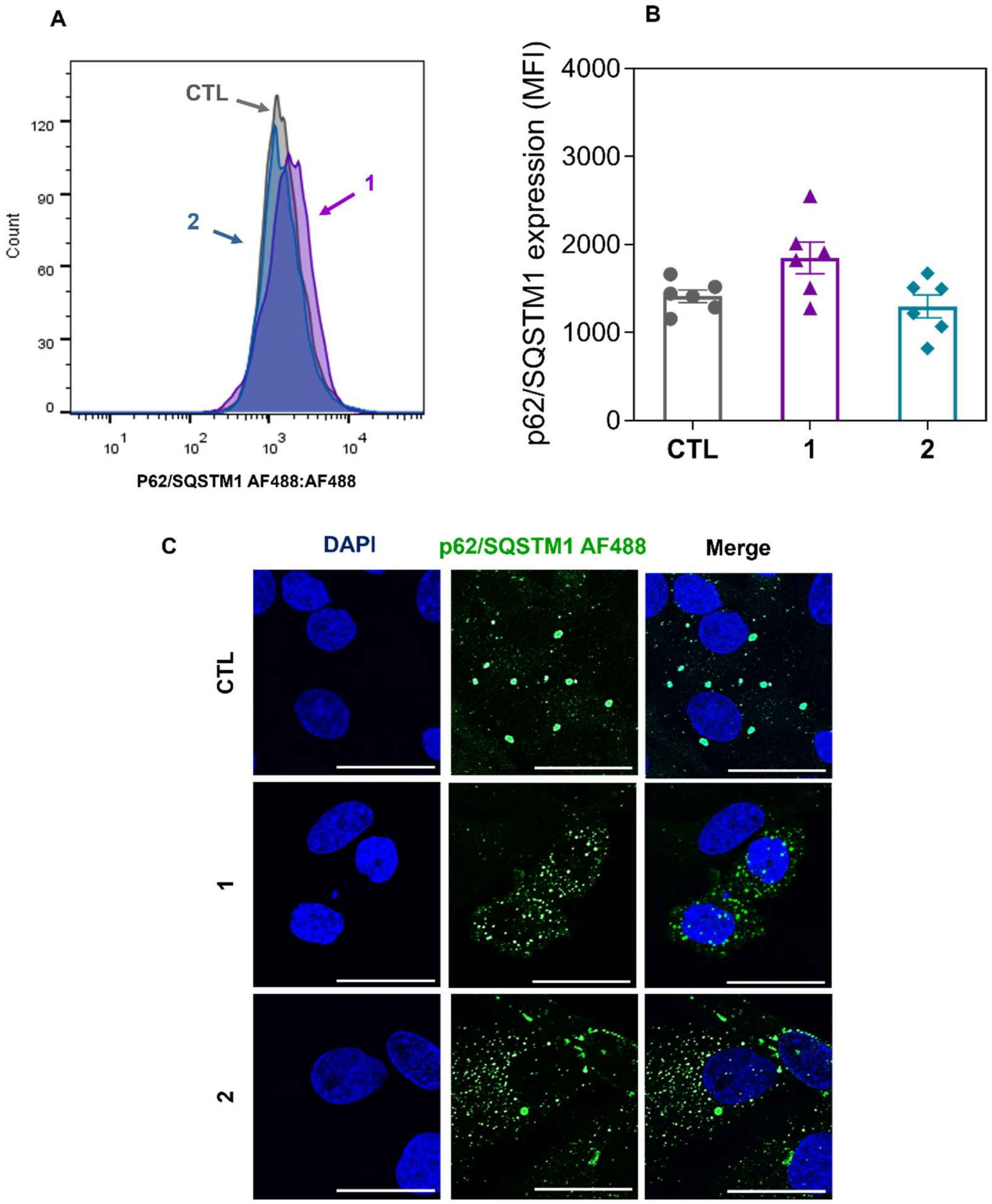

Figure S8. Quantification of p62/SQSTM1 expression (**A** and **B**) in HepG2 cells after 24 h of incubation with 8 μM complex **1** or 6 μM complex **2**, as determined by flow cytometric analysis. The vehicle (0.2% DMSO) was used as a negative control (CTL). The data are expressed as the mean ± S.E.M. of three biological replicates carried out in duplicate. MFI: Mean fluorescence intensity. (**C**) Representative immunofluorescence images of p62/SQSTM1 in HepG2 cells after 24 h of incubation with 8 μM complex **1** or 6 μM complex **2**. Scale bar = 25 μm.

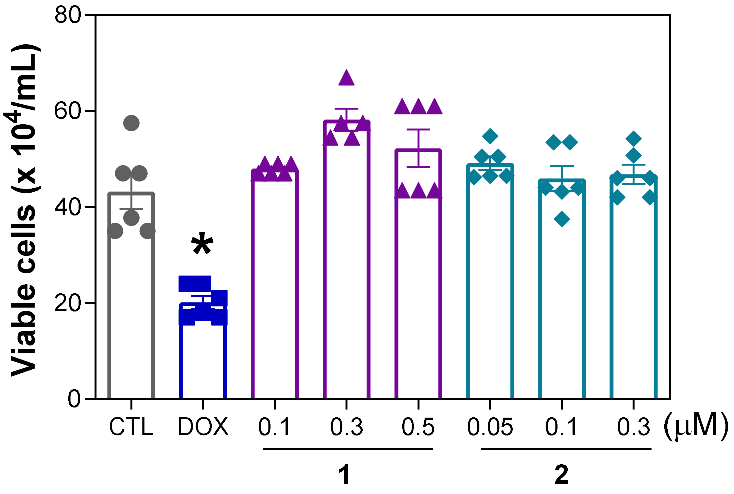

Figure S9. Quantification of viable HepG2 cells after 72 h of incubation with complexes **1** and **2**, as determined by the trypan blue method. The vehicle (0.2% DMSO) was used as a negative control (CTL), and doxorubicin (DOX, 1 μM) was used as a positive control. The data are expressed as the mean ± S.E.M. of three biological replicates carried out in duplicate. * *P* < 0.05 compared to CTL by one-way analysis of variance (ANOVA) followed by Dunnett's multiple comparisons test.

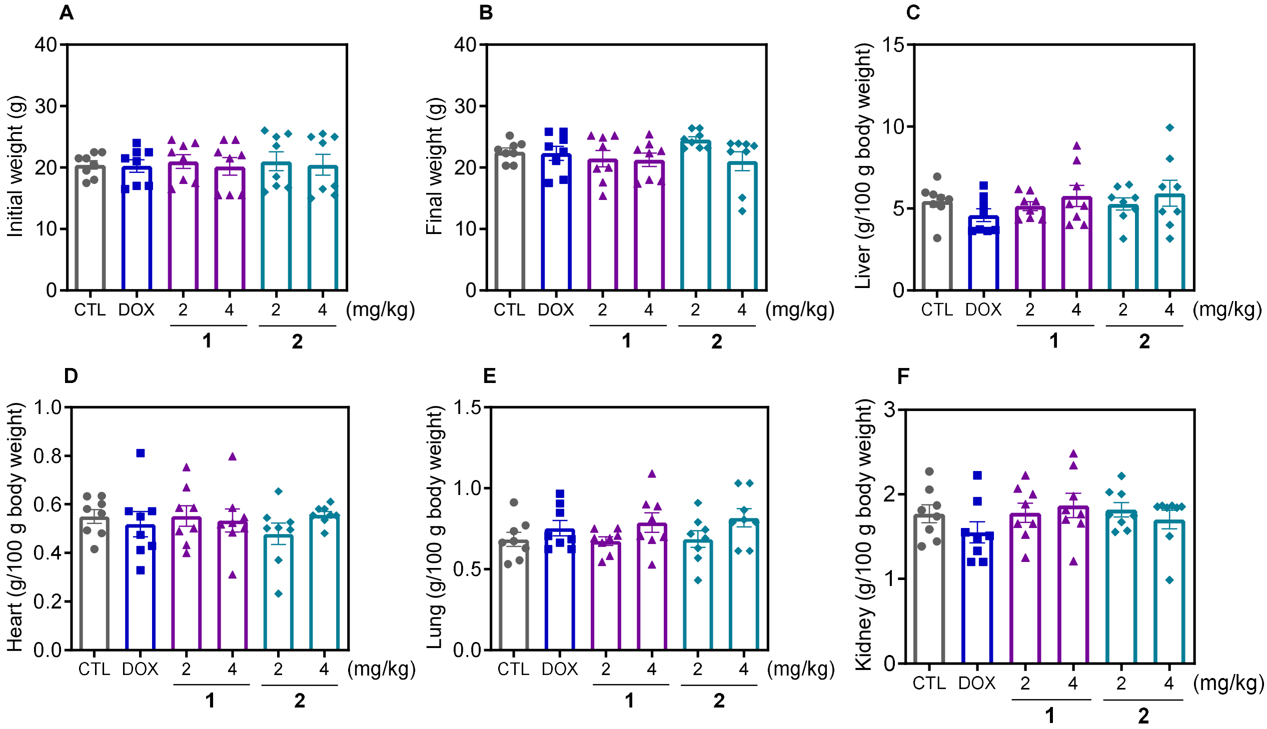

Figure S10. Effect of treatment with complexes **1** and **2** on the body weight (**A** and **B**) and relative organ weight (**C**, **D**, **E** and **F**) of C.B-17 SCID mice inoculated with HepG2 cells. The vehicle (5% DMSO) was used as a negative control (CTL), and doxorubicin (DOX, 1 mg/kg) was used as a positive control. The data are expressed as the mean ± S.E.M. from 8 animals.

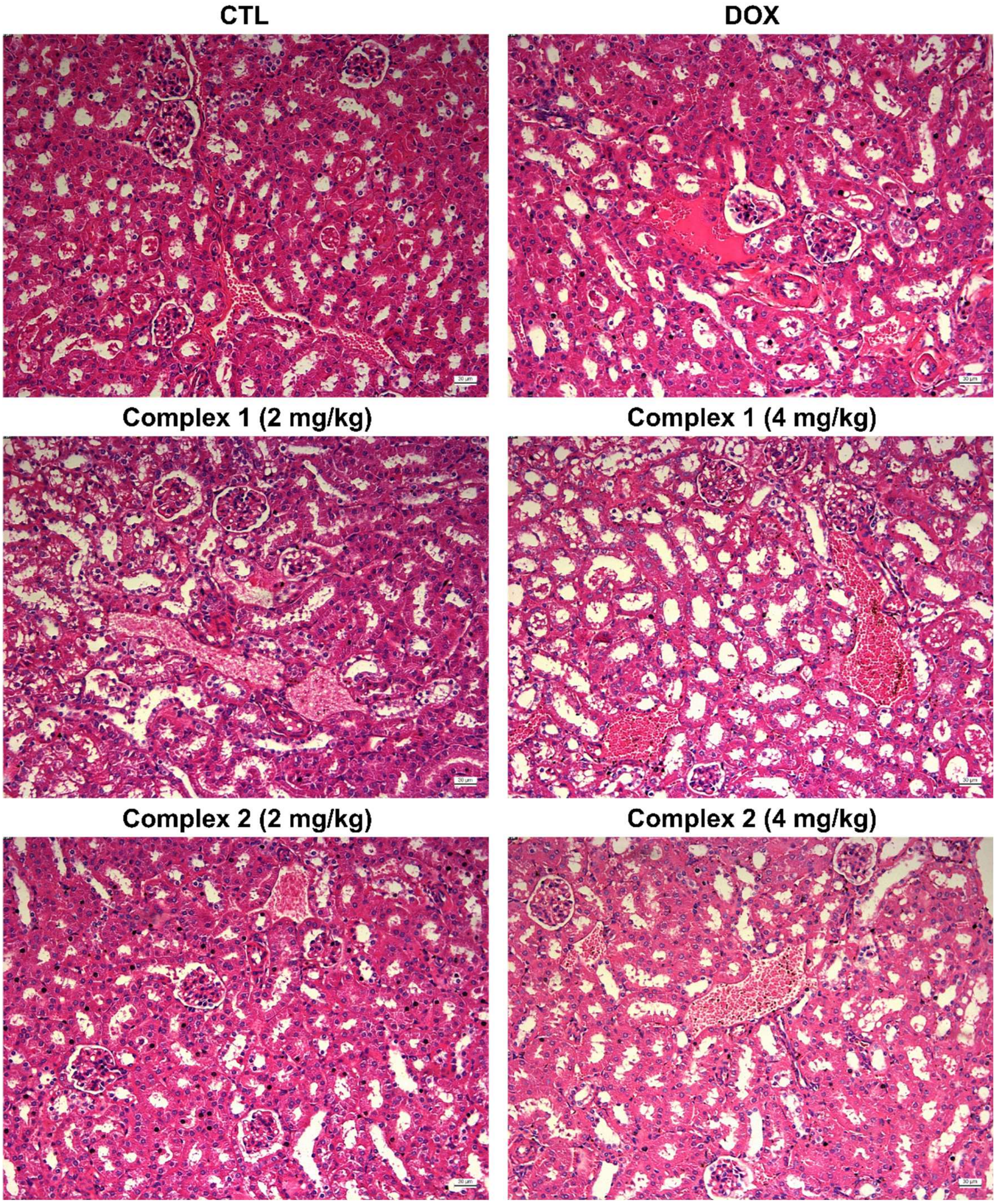

Figure S11. Representative photomicrographs of the kidneys of animals treated with complexes 1 and 2. Histological sections were stained with hematoxylin-eosin and analyzed by light microscopy. The vehicle (5% DMSO) was used as a negative control (CTL), and doxorubicin (DOX, 1 mg/kg) was used as a positive control. The renal parenchyma was preserved in all animals; however, focal areas of coagulation necrosis of the renal cortex tubules were observed. Among the morphological changes observed in this organ, vascular dilation and hyperemia, which ranged from moderate to severe, and a slight decrease in Bowman's space due to glomerular hyalinization were observed. It is important to emphasize that these alterations were more evident in the groups treated with DOX and the higher doses of complexes **1** and **2**.

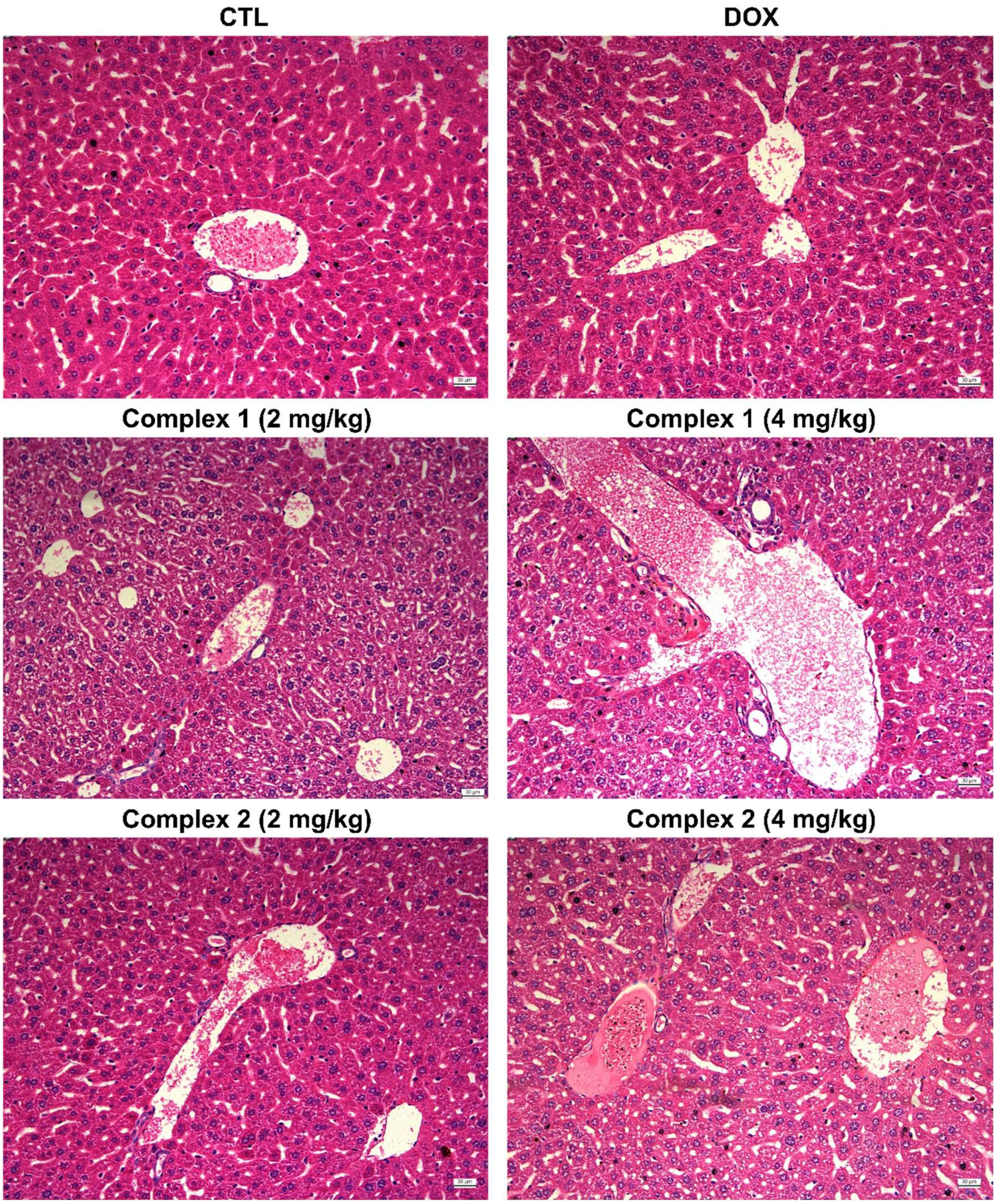

Figure S12. Representative photomicrographs of the livers of animals treated with complexes 1 and 2. Histological sections were stained with hematoxylin-eosin and analyzed by light microscopy. The vehicle (5% DMSO) was used as a negative control (CTL), and doxorubicin (DOX, 1 mg/kg) was used as a positive control. The portal architecture ranged from preserved to partially preserved in the livers of all the experimental groups, with vascular dilation and hyperemia being more evident in the venules of the animals treated with DOX and complexes **1** and **2**. The liver parenchyma was partially preserved in most animals due to the presence of hydropic degeneration, which varied from mild to moderate, and focal areas of coagulation necrosis. Furthermore, moderate vascular hyperemia and focal areas of mixed inflammation were observed, with a predominance of polymorphonuclear cells.

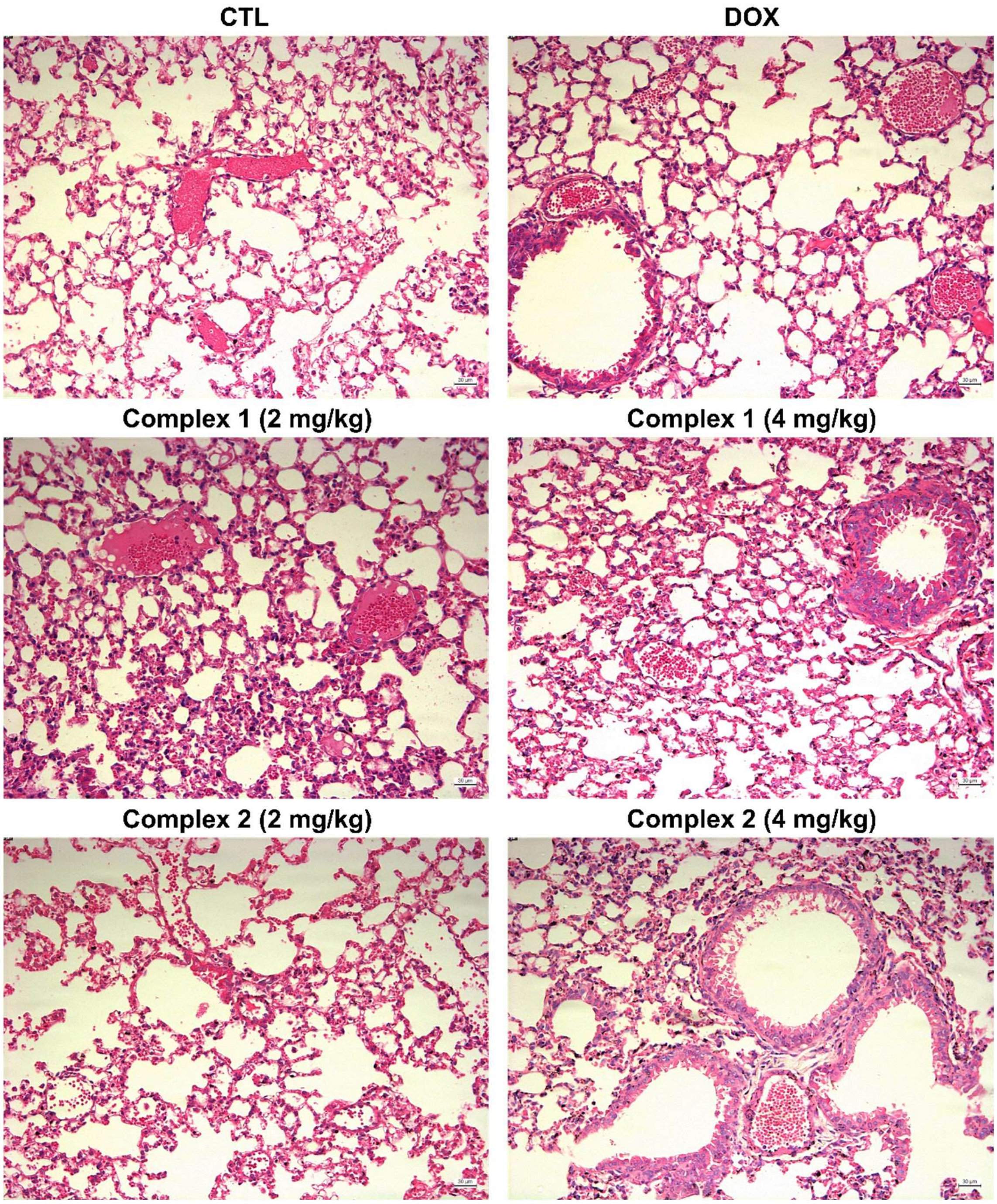

Figure S13. Representative photomicrographs of the lungs of animals treated with complexes 1 and 2. Histological sections were stained with hematoxylin-eosin and analyzed by light microscopy. The vehicle (5% DMSO) was used as a negative control (CTL), and doxorubicin (DOX, 1 mg/kg) was used as a positive control. The architecture of the lung parenchyma ranged from preserved to partially preserved, and this alteration was mainly related to thickening of the alveolar septa and, consequently, pulmonary atelectasis. Histopathological changes such as vasodilation, vascular hyperemia, polymorphonuclear cell infiltration, edema, hemorrhage, and focal areas of hemosiderin deposition were observed in all the animals and ranged from mild to severe.

Table S1. IC_{50} values against cancer and noncancerous cells

Cells	IC_{50} and 95% confidence interval (in µM)		
	1	2	DOX
Cancer cells			
HepG2	3.49	3.17	0.32
	2.14 – 5.70	2.23 – 4.50	0.13 – 0.78
HCT116	4.74	4.52	0.86
	3.05 – 7.36	3.14 – 6.49	0.50 – 1.48
NB4	4.79	5.25	0.09
	2.29 – 9.87	2.77 – 10.67	0.05 – 0.13
THP-1	4.75	6.47	0.14
	3.66 – 6.18	4.65 – 8.98	0.10 – 0.21
Jurkat	5.07	4.94	0.06
	4.49 – 5.72	4.45 – 5.48	0.03 – 0.10
K-562	6.77	7.69	1.65
	4.73 – 9.69	5.42 – 10.90	0.85 – 3.21
HL-60	5.71	4.11	0.35
	4.74 – 6.88	3.66 – 4.62	0.32 – 0.39
KG-1a	4.77	3.77	0.07
	3.40 – 6.68	2.41 – 5.77	0.02 – 0.33
MDA-MB-231	2.62	2.95	0.43
	1.98 – 3.46	2.30 – 3.79	0.15 – 1.21
MCF-7	9.21	7.29	2.28
	5.92 – 15.46	4.66 – 11.01	1.59 – 3.46

4T1	11.72<br>9.28 – 14.79	4.52<br>3.74 – 5.46	1.53<br>1.17 – 2.01
HSC-3	8.36<br>6.16 – 11.57	5.55<br>4.08 – 7.47	0.69<br>0.49 – 0.99
CAL 27	2.66<br>1.67 – 4.07	4.31<br>2.66 – 6.65	0.11<br>0.08 – 0.27
SCC-25	7.61<br>5.28 – 10.95	3.63<br>2.77 – 4.75	1.44<br>1.02 – 2.05
SCC4	4.77<br>4.02 – 5.69	3.48<br>3.18 – 3.80	1.80<br>1.55 – 2.10
SCC-9	4.40<br>2.92 – 6.64	2.54<br>1.84 – 3.51	1.00<br>0.62 – 1.63
A549	17.50<br>12.17 – 25.17	10.46<br>8.32 – 13.14	2.35<br>1.95 – 2.84
PANC-1	9.45<br>6.29 – 14.19	4.09<br>3.09 – 5.42	1.11<br>0.74 – 1.67
OVCAR-3	2.43<br>2.00 - 2,95	1.62<br>1.26 – 2.08	1.06<br>0.65 – 1.74
DU 145	7.60<br>5.51 – 10.48	3.74<br>2.95 – 4.75	0.24<br>0.17 – 0.35
U-87 MG	3.53<br>2.54 – 4.90	3.69<br>2.78 – 4.91	0.52<br>0.33 – 0.80
A-375	3.01<br>2.44 – 3.73	2.00<br>1.64 – 2.44	0.16<br>0.13 – 0.20
B16-F10	3.04	2.75	0.43

	2.47 – 3.73	2.31 – 3.28	0.29 – 0.65
Noncancerous cells			
PBMC	14.23	11.66	1.33
	12.28 – 16.51	9.89 – 13.74	0.93 – 1.88
MRC-5	17.70	15.22	1.70
	12.60 – 24.87	11.07 – 20.94	0.87 – 3.32
BJ	7.00	7.79	1.85
	4.07 – 14.37	6.11 – 10.18	1.08 – 3.81

These data were obtained by nonlinear regression from biological replicates carried out in duplicate after 72 h of incubation, as determined by the Alamar Blue assay. Doxorubicin (DOX) was used as a positive control.

Table S2. Calculated selectivity indices

Cancer cells	Noncancerous cells								
	PBMC			MRC-5			BJ		
	1	2	DOX	1	2	DOX	1	2	DOX
HepG2	4.08	3.68	4.16	5.07	4.80	5.31	1.72	2.46	5.78
HCT116	3.00	2.58	1.55	3.73	3.37	1.98	2.33	1.72	2.15
NB4	2.97	2.22	14.78	3.70	2.90	18.89	2.36	1.48	20.56
THP-1	3.00	1.80	9.50	3.73	2.35	12.14	2.34	1.20	13.21
Jurkat	2.81	2.36	22.17	3.49	3.08	28.33	2.49	1.58	30.83
K-562	2.10	1.52	0.81	2.61	1.98	1.03	3.33	1.01	1.12
HL-60	2.49	2.84	3.80	3.10	3.70	4.86	2.81	1.90	5.29
KG-1a	2.98	3.09	19.00	3.71	4.04	24.29	2.35	2.07	26.43
MDA-MB-231	5.43	3.95	3.09	6.76	5.16	3.95	1.29	2.64	4.30
MCF-7	1.55	1.60	0.58	1.92	2.09	0.75	4.53	1.07	0.81
4T1	1.21	2.58	0.87	1.51	3.37	1.11	5.77	1.72	1.21
HSC-3	1.70	2.10	1.93	2.12	2.74	2.46	4.11	1.40	2.68
CAL 27	5.35	2.71	12.09	6.65	3.53	15.45	1.31	1.81	16.82
SCC-25	1.87	3.21	0.92	2.33	4.19	1.18	3.74	2.15	1.28
SCC4	2.98	3.35	0.74	3.71	4.37	0.94	2.35	2.24	1.03
SCC-9	3.23	4.59	1.33	4.02	5.99	1.70	2.16	3.07	1.85
A549	0.81	1.11	0.57	1.01	1.46	0.72	8.61	0.74	0.79
PANC-1	1.51	2.85	1.20	1.87	3.72	1.53	4.65	1.90	1.67
OVCAR-3	5.86	7.20	1.25	7.28	9.40	1.60	1.20	4.81	1.75

DU 145	1.87	3.12	5.54	2.33	4.07	7.08	3.74	2.08	7.71
U-87 MG	4.03	3.16	2.56	5.01	4.12	3.27	1.74	2.11	3.56
A-375	4.73	5.83	8.31	5.88	7.61	10.63	1.48	3.90	11.56
B16-F10	4.68	4.24	3.09	5.82	5.53	3.95	1.50	2.83	4.30

The data were calculated using the following formula: selectivity indices = IC_{50} [noncancerous cells]/IC_{50} [cancer cells].

Table S3. The effect of complexes **1** and **2** on gene expression in HepG2 cells

**Function/Assay ID**	**Gene symbol**	**Full name**	**RQ**		
			**CTL**	**1**	**2**
**Apoptosis**					
Hs00608023_m1	*BCL2*	BCL2, apoptosis regulator	1.00	1.602	1.249
Hs04194392_s1	*BIRC5*	baculoviral IAP repeat containing 5	1.00	0.800	1.033
**PI3 Kinases & Phosphatases**					
Hs00234508_m1	*MTOR*	mechanistic target of rapamycin	1.00	0.395	1.092
Hs00904054_m1	*PIK3C2A*	phosphatidylinositol-4-phosphate 3-kinase catalytic subunit type 2 alpha	1.00	0.855	0.839
Hs00176908_m1	*PIK3C3*	phosphatidylinositol 3-kinase catalytic subunit type 3	1.00	1.001	0.840
Hs00907957_m1	*PIK3CA*	phosphatidylinositol-4,5-bisphosphate 3-kinase catalytic subunit alpha	1.00	0.817	0.441
**Growth Factors & Receptors**					
Hs01076090_m1	*EGFR*	epidermal growth factor receptor	1.00	1.217	1.856
Hs01001580_m1	*ERBB2*	erb-b2 receptor tyrosine kinase 2	1.00	0.682	1.118
Hs00176538_m1	*ERBB3*	erb-b2 receptor tyrosine kinase 3	1.00	0.915	1.280

Hs01128657_m1	*FIGF*	c-fos induced growth factor	1.00	N.d.	N.d.
Hs01052961_m1	*FLT1*	fms related tyrosine kinase 1	N.d.	N.d.	N.d.
Hs01047677_m1	*FLT4*	fms related tyrosine kinase 4	N.d.	N.d.	N.d.
Hs01547656_m1	*IGF1*	insulin like growth factor 1	N.d.	N.d.	N.d.
Hs00609566_m1	*IGF1R*	insulin like growth factor 1 receptor	1.00	0.916	1.357
Hs04188276_m1	*IGF2*	insulin like growth factor 2	N.d.	N.d.	N.d.
Hs00911700_m1	*KDR*	kinase insert domain receptor	N.d.	N.d.	N.d.
Hs00174029_m1	*KIT*	KIT proto-oncogene receptor tyrosine kinase	N.d.	N.d.	N.d.
Hs00998018_m1	*PDGFRA*	platelet derived growth factor receptor alpha	N.d.	N.d.	N.d.
Hs01019589_m1	*PDGFRB*	platelet derived growth factor receptor beta	1.00	0.964	1.004
**Drug Metabolism**					
Hs01561483_m1	*ABCC1*	ATP binding cassette subfamily C member 1	1.00	2.226	1.941
Hs00943350_g1	*GSTP1*	glutathione S-transferase pi 1	1.00	0.499	0.276
Hs00153133_m1	*PTGS2*	prostaglandin-endoperoxide synthase 2	1.00	7.499	15.420
Hs01555214_g1	*TXN*	thioredoxin	1.00	0.546	0.375
Hs00917067_m1	*TXNRD1*	thioredoxin reductase 1	1.00	1.341	0.350
**G-Protein Signaling**					

Hs00357608_m1	*RHOA*	ras homolog family member A	1.00	0.642	0.628
Hs03676562_s1	*RHOB*	ras homolog family member B	1.00	0.437	0.406
**Hormone Receptors**					
Hs01046816_m1	*ESR1*	estrogen receptor 1	1.00	0.564	0.968
Hs01100353_m1	*ESR2*	estrogen receptor 2	1.00	2.165	2.732
Hs01556702_m1	*PGR*	progesterone receptor	1.00	8.929	30.650
**Heat Shock Proteins**					
Hs00743767_sH	*HSP90AA1*	heat shock protein 90 alpha family class A member 1	1.00	2.760	3.278
Hs00427665_g1	*HSP90B1*	heat shock protein 90 beta family member 1	1.00	3.676	2.205
**Receptor Tyrosine Kinase Signaling**					
Hs00178289_m1	*AKT1*	AKT serine/threonine kinase 1	1.00	0.775	1.087
Hs01086099_m1	*AKT2*	AKT serine/threonine kinase 2	1.00	0.889	1.490
Hs00157817_m1	*GRB2*	growth factor receptor bound protein 2	1.00	1.501	2.255
**Cathepsins**					
Hs00947439_m1	*CTSB*	cathepsin B	1.00	0.797	0.972
Hs00157205_m1	*CTSD*	cathepsin D	1.00	1.503	1.586
Hs00964650_m1	*CTSL*	cathepsin L	1.00	1.271	0.950
Hs00175407_m1	*CTSS*	cathepsin S	1.00	1.314	0.866
**Cell Cycle**					

Hs00947994_m1	*CDC25A*	cell division cycle 25A	1.00	2.454	6.141
Hs00938777_m1	*CDK1*	cyclin dependent kinase 1	1.00	0.664	0.752
Hs01548894_m1	*CDK2*	cyclin dependent kinase 2	1.00	0.777	1.120
Hs00364847_m1	*CDK4*	cyclin dependent kinase 4	1.00	1.130	1.730
Hs00358991_g1	*CDK5*	cyclin dependent kinase 5	1.00	0.572	1.104
Hs00361486_m1	*CDK7*	cyclin dependent kinase 7	1.00	2.521	2.039
Hs00992501_g1	*CDK8*	cyclin dependent kinase 8	1.00	2.592	3.664
Hs00977896_g1	*CDK9*	cyclin dependent kinase 9	1.00	0.937	1.146
Hs00540450_s1	*MDM2*	MDM2 proto-oncogene	1.00	1.685	0.982
Hs00967238_m1	*MDM4*	MDM4, p53 regulator	1.00	2.263	2.736
Hs00972650_m1	*TERT*	telomerase reverse transcriptase	1.00	0.604	1.051
**Topoisomerases, Type II**					
Hs01032137_m1	*TOP2A*	topoisomerase (DNA) II alpha	1.00	0.469	0.423
Hs00172259_m1	*TOP2B*	topoisomerase (DNA) II beta	1.00	0.499	0.235
**Transcription Factors**					
Hs01095345_m1	*ATF2*	activating transcription factor 2	1.00	1.702	2.672
Hs00153153_m1	*HIF1A*	hypoxia inducible factor 1 alpha subunit	1.00	3.170	3.509
Hs00158114_m1	*IRF5*	interferon regulatory factor 5	N.d.	N.d.	N.d.
Hs00765730_m1	*NFKB1*	nuclear factor kappa B subunit 1	1.00	0.451	0.760

Hs01034249_m1	*TP53*	tumor protein p53	1.00	1.021	1.658
**Protein Kinases**					
Hs01582072_m1	*AURKA*	aurora kinase A	1.00	0.691	1.172
Hs00945858_g1	*AURKB*	aurora kinase B	1.00	0.759	1.242
Hs00152930_m1	*AURKC*	aurora kinase C	1.00	1.565	2.011
Hs00983227_m1	*PLK1*	polo like kinase 1	1.00	0.520	0.509
Hs00198320_m1	*PLK2*	polo like kinase 2	1.00	1.630	5.868
Hs00177725_m1	*PLK3*	polo like kinase 3	1.00	2.378	2.039
Hs00179514_m1	*PLK4*	polo like kinase 4	1.00	0.743	0.844
Hs00925200_m1	*PRKCA*	protein kinase C alpha	1.00	1.667	1.337
Hs00176998_m1	*PRKCB*	protein kinase C beta	1.00	0.327	0.506
Hs01090047_m1	*PRKCD*	protein kinase C delta	1.00	0.661	0.340
Hs00942886_m1	*PRKCE*	protein kinase C epsilon	1.00	2.615	1.636
**RAS Signaling**					
Hs00978050_g1	*HRAS*	HRas proto-oncogene, GTPase	1.00	0.753	0.785
Hs00364284_g1	*KRAS*	KRas proto-oncogene, GTPase	1.00	1.809	1.185
Hs00180035_m1	*NRAS*	NRas proto-oncogene, GTPase	1.00	0.908	0.469
**Histone Deacetylases**					
Hs00978031_g1	*HDAC1*	histone deacetylase 1	1.00	0.833	16.128
Hs00978031_g1	*HDAC11*	histone deacetylase 11	1.00	1.729	3.379

Hs00231032_m1	*HDAC2*	histone deacetylase 2	1.00	1.190	2.118
Hs00187320_m1	*HDAC3*	histone deacetylase 3	1.00	0.989	3.639
Hs01041648_m1	*HDAC4*	histone deacetylase 4	1.00	0.764	0.456
Hs00997427_m1	*HDAC6*	histone deacetylase 6	1.00	0.564	1.088
Hs01045864_m1	*HDAC7*	histone deacetylase 7	1.00	1.008	2.228
Hs00954353_g1	*HDAC8*	histone deacetylase 8	1.00	0.970	1.517
**Poly ADP-Ribose Polymerases**					
Hs00242302_m1	*PARP1*	poly(ADP-ribose) polymerase 1	1.00	0.638	1.065
Hs00173105_m1	*PARP4*	poly(ADP-ribose) polymerase family member 4	1.00	0.801	2.503
Hs00186671_m1	*TNKS*	tankyrase	1.00	3.189	2.369
**Structural Proteins**					
Hs00362403_g1	*NTN3*	netrin 3	1.00	5.849	0.712

HepG2 cells were treated with 8 μM complex **1** or 6 μM complex **2** for 12 h. Vehicle (0.2% DMSO) was used as a negative control (CTL). After treatment, total RNA was isolated and reverse transcribed. Gene expression was detected using a TaqMan® Array Human Cancer Drug Targets 96-well plate. The *GAPDH*, *B2M* and *RPLP0* genes were used as endogenous genes for normalization. The values represent the relative quantitation (RQ) compared with the calibrator (cells treated with the negative control). The genes were upregulated if RQ ≥ 2 and downregulated if RQ ≤ 0.5. N.d. Not determined.

Table S4. List of cells used

Cells	Histological type	Species	Source[a,b]
Cancer cell lines			
HepG2	hepatocellular carcinoma	human	ATCC
HCT116	colorectal carcinoma	human	ATCC
NB4	acute promyelocytic leukemia	human	ATCC
THP-1	monocytic leukemia	human	ATCC
Jurkat	T-cell lymphoid leukemia	human	ATCC
K-562	chronic myelogenous leukemia	human	ATCC
HL-60	acute promyelocytic leukemia	human	ATCC
KG-1a	acute myeloid leukemia	human	ATCC
MDA-MB-231	breast carcinoma	human	BCRJ
MCF-7	breast adenocarcinoma	human	ATCC
4T1	breast carcinoma	mouse	ATCC
HSC-3	oral squamous cell carcinoma	human	ATCC
CAL 27	oral squamous cell carcinoma	human	ATCC
SCC-25	oral squamous cell carcinoma	human	ATCC
SCC4	oral squamous cell carcinoma	human	ATCC
SCC-9	oral squamous cell carcinoma	human	ATCC
A549	lung adenocarcinoma	human	BCRJ
PANC-1	pancreas ductal adenocarcinoma	human	BCRJ
OVCAR-3	ovarian carcinoma	human	BCRJ
DU 145	prostate carcinoma	human	BCRJ
U-87 MG	glioblastoma	human	BCRJ

A-375	melanoma	human	BCRJ
B16-F10	melanoma	mouse	ATCC
Noncancer cell lines			
PBMC	heath peripheral blood mononuclear cells	human	primary cell culture
MRC-5	lung fibroblast	human	ATCC
BJ	foreskin fibroblast	human	ATCC
Mutant and its parental cell lines			
BAD KO SV40 MEF	immortalized mouse embryonic fibroblasts with the BAD gene knocked out	mouse	ATCC
WT SV40 MEF	wild-type immortalized embryonic fibroblasts	mouse	ATCC

[a]ATCC denotes the American Type Culture Collection (U.S.A.), and BCRJ denotes the Rio de Janeiro Cell Bank (Brazil). [b]Primary cell culture of PBMCs was obtained from peripheral blood from healthy donors by a standard Ficoll density protocol. Then, the PBMCs were resuspended in RPMI 1640 or DMEM-F12 supplemented with 20% FBS and 1% antibiotics. The cells were plated at 5×10^5 cells/well. Concanavalin A (10 µg/mL, Sigma–Aldrich) was used as a mitogen to trigger cell division in T lymphocytes and was added at the beginning of the culture. The Research Ethics Committee of the Oswaldo Cruz Foundation (Salvador, Bahia, Brazil) approved the protocol (CAAE 16220713.2.0000.0040).

Table S5. List of antibodies used

Epitope	Fluorochrome	Clone	Application	Catalog number	Company
Akt 1	PE	55/PKBa/Akt	Intracellular staining	560049	BD Phosflow
CD133	PE	W6B3C1 (W6B3)	Cell surface staining	566594	BD Pharmingen
CD44	BV421	G44-26	Cell surface staining	562890	BD Horizon™
Cleaved PARP (Asp214)	PE	F21-852	Intracellular staining	552933	BD Pharmingen
E-cadherin	PE	67A4	Cell surface staining	562870	BD Pharmingen™
IgG1, κ Isotype Control	PE	MOPC-21	Cell surface staining	556650	BD Pharmingen
p62/SQSTM1	AF488	864807	Intracellular staining	IC8028G	R&D Systems
Phospho-4EBP1 (T36/T45)	AF488	M31-16	Intracellular staining	560287	BD Phosflow
Phospho-Akt (S473)	AF488	M89-61	Intracellular staining	560404	BD Phosflow
Phospho-Akt (T308)	PE	J1-223.371	Intracellular staining	558275	BD Phosflow

Phospho-eIF4E (S209)	PE	J77-925	Intracellular staining	560229	BD Phosflow
Phospho-mTOR (S2448)	PE	O21-404	Intracellular staining	563489	BD Phosflow
Phospho-NF-κB p65 (S529)	AF488	K10-895.12.50	Intracellular staining	558421	BD Phosflow
Phospho-PI3K p85/p55 (T458/T199)	PE	PI3KY458-1A11	Intracellular staining	MAS-28027	Invitrogen
Phospho-S6 (S235/S236)	AF488	N7-548	Intracellular staining	560434	BD Phosflow
Vimentin	AF488	RV202	Intracellular staining	562338	BD Pharmingen
